# The Divergence of Flowering Time Modulated by *FT/TFL1* Is Independent to Their Interaction and Binding Activities

**DOI:** 10.3389/fpls.2017.00697

**Published:** 2017-05-08

**Authors:** Zhen Wang, Ruiguang Yang, Upendra K. Devisetty, Julin N. Maloof, Yang Zuo, Jingjing Li, Yuxiao Shen, Jian Zhao, Manzhu Bao, Guogui Ning

**Affiliations:** ^1^Key Laboratory of Horticultural Plant Biology, Ministry of Education, College of Horticulture and Forestry Sciences, Huazhong Agricultural UniversityWuhan, China; ^2^BIO5 Institute, University of Arizona, TucsonAZ, USA; ^3^Department of Plant Biology, University of California, Davis, DavisCA, USA; ^4^National Key Laboratory of Crop Genetics and Improvement, College of Plant Science and Technology, Huazhong Agricultural UniversityWuhan, China

**Keywords:** *FT/TFL1* homologs, site mutated, transgenic research, protein interactions, binding activity, Rosaceae species

## Abstract

FLOWERING LOCUS T (*FT*) and TERMINAL FLOWER1 (*TFL1*) proteins share highly conserved amino acid residues but they play opposite regulatory roles in promoting and repressing the flowering response, respectively. Previous substitution models and functional analysis have identified several key amino acid residues which are critical for the promotion of flowering. However, the precise relationship between naturally occurring *FT/TFL1* homologs and the mechanism of their role in flowering is still unclear. In this study, *FT/TFL1* homologs from eight Rosaceae species, namely, *Spiraea cantoniensis, Pyracantha fortuneana, Photinia serrulata, Fragaria ananassa, Rosa hybrida, Prunus mume, Prunus persica* and *Prunus yedoensis*, were isolated. Three of these homologs were further characterized by functional analyses involving site-directed mutagenesis. The results showed that these *FT/TFL1* homologs might have diverse functions despite sharing a high similarity of sequences or crystal structures. Functional analyses were conducted for the key FT amino acids, Tyr-85 and Gln-140. It revealed that *TFL1* homologs cannot promote flowering simply by substitution with key *FT* amino acid residues. Mutations of the IYN triplet motif within segment C of exon 4 can prevent the *FT* homolog from promoting the flowering. Furthermore, physical interaction of FT homologous or mutated proteins with the transcription factor FD, together with their lipid-binding properties analysis, showed that it was not sufficient to trigger flowering. Thus, our findings revealed that the divergence of flowering time modulating by *FT/TFL1* homologs is independent to interaction and binding activities.

## Introduction

Flowering is a key developmental phase of the higher plants. The transition from the vegetative to reproductive growth phase is tightly regulated by a complex arrangement of multiple signaling networks. In *Arabidopsis thaliana*, multiple regulatory pathways involved in the flowering have been thoroughly researched. Generally it includes photoperiod, vernalization, hormone, autonomous, and age-dependent pathways (Mutasa-Göttgens and Hedden, 2009; Turnbull, 2011; Wang R.H. et al., 2011; Johansson and Staiger, 2015; Wagner, 2016). These multiple pathways converge upon a small set of key flowering time genes which are responsible for growth phase transition and the onset of flowering. The mobile florigen *FLOWERING LOCUS T* (*FT*), *SUPRESSOR OF OVEREXPRESSION OF CONSTANS1* (*SOC1*) and *LEAFY* (*LFY*) genes function as integrators of different regulatory pathways.

*FT* and *FT*-homologs are floral promoter genes and they are highly conserved in a wide range of plant species (Coelho et al., 2014; Xing et al., 2014; Wolabu et al., 2016). Current understanding is that the *FT* gene is expressed within the leaves, while the mature protein moves to the shoot apex via the phloem, where it interacts with FD to participate in the promotion of flowering (Wigge et al., 2005; Notaguchi et al., 2008; Benlloch et al., 2011). Thus, *FT* had been extensively studied as a candidate for the mobile flower-promoting signal known as “florigen” (Corbesier et al., 2007; Kobayashi and Weigel, 2007; Tamaki et al., 2007). Conversely, flowering is strongly repressed by the *FT* homolog, *TFL1* (Bradley et al., 1997; Ohshima et al., 1997). In *Arabidopsis, TFL1* has been proposed to repress flowering both by antagonizing the activity of *FT* and also through an independent flowering control activity (Kardailsky et al., 1999; Kobayashi et al., 1999; Pnueli et al., 2001).

*FT* and *TFL1* encode proteins approximately 175 amino acids and their structure is similar to a phosphatidylethanolamine-binding protein (PEBP) family found in mammalian, yeast, and bacteria (Grandy et al., 1990; Bradley et al., 1996). PEBPs have been shown to act in multiple roles as modulators in cell growth and differentiation (Hengst et al., 2001; Fu et al., 2003; Chautard et al., 2004). Plant PEBP-related genes were initially cloned from *Antirrhinum* (Bradley et al., 1996), *Arabidopsis* (Bradley et al., 1997) and tomato (Pnueli et al., 1998). The structure of each of these proteins have now also been illustrated (Banfield and Brady, 2000; Ahn et al., 2006). It revealed that the tertiary structures of the plant PEBPs are also closely similar to those of animal counterparts, being dominated by a large central β-sheet with an anion binding pocket contacted by a C-terminal peptide. However, there is no direct evidence in the plant PEBPs that phospholipids or other anions binding to this pocket *in vivo*, as seen in the animal PEBPs (Banfield et al., 1998; Serre et al., 1998; Simister et al., 2002). The phospholipid binding activity test showed that FT bound to the lipid phosphatidylcholine (PC) *in vitro*, but not to phosphatidylethanolamine (PE). It was partially related to FT activity since the ratio of PC: PE increasing accelerates flowering (Nakamura et al., 2014).

FT and TFL1 play opposing roles in the control of flowering, though there are only 39 non-conservative residues between them in *Arabidopsis* (Ho and Weigel, 2014). Thus, the question is arisen whether certain critical residues are responsible for the diversity of their functions. It has been reported that Tyr-85 in FT and His-88 in TFL1 play key roles in their respective functions. Substitution of the amino acid residues at these positions (i.e., replacing Tyr to His in FT, or His to Tyr in TFL1) was found to confer partial TFL1-like activity on the altered FT protein and weak FT-like activity on the altered form of TFL1 (Hanzawa et al., 2005). *Arabidopsis* demonstrated an early flowering phenotype when an *OnTFL1* orchid homolog H85Y was ectopically expressed (Hou and Yang, 2009). Subsequent experiments showed an external loop structure (residues 128–145), together with the adjacent peptide segment, contributed to the opposite FT and TFL1 activities (Ahn et al., 2006). The external loop segment is almost invariant in FT orthologs, but it seems to have evolved rapidly in TFL1 orthologs. Furthermore, a specific residue in this external loop structure makes a hydrogen bond with His-88 near to the entrance of a potential ligand-binding pocket in TFL1, but not in FT (Hanzawa et al., 2005; Ahn et al., 2006; Ho and Weigel, 2014). In sugar beet (*Beta vulgaris* subsp. *vulgaris*), two paralogs of *FT* (i.e., *BvFT1* and *BvFT2*) both contain Tyr-85 and Gln-140, but they have naturally evolved antagonistic functions. Whereas BvFT2 is essential for flowering, BvFT1 acts as a flowering repressor. In *BvFT1* it was shown that the alteration of three amino acids in the external loop structure could reverse its repressor function into a floral promotion role (Pin et al., 2010). Ho and Weigel (2014) found that specific mutations at the four Glu-109, Trp-138, Gln-140, and Asn-152 residues could transform FT into a TFL1-like floral repressor.

Here, we report the isolation and characterization of the *FT/TFL1* homologs of eight Rosaceae species. Ectopic overexpression analysis of various *FT/TFL1* homologs showed that there was a diversity function among them in spite of the high levels of similarity. Site mutation analysis of selected *FT/TFL1* homologs identified a specific amino acid residue (N-154 of RoFT), not previously reported, to be important to the maintenance of floral promoting. Interaction analysis between AtFD and the phenotype specific FT/TFL1 homologs or mutations indicated that FT homologs in flowering promotion are not a simple function of the interaction with FD. In addition, the putative phospholipid binding investigations shown that all of flowering promoted or delayed FT/TFL1 homologs or mutations have the same lipid-binding properties. Our findings provide evidence that the diversity of flowering time modulating by FT/TFL1 homologs is independent to their interaction and binding activities.

## Materials and Methods

### Plant Materials

Plants of eight Rosaceae species were from the experimental plots at Huazhong Agricultural University, Wuhan, P.R. China. *Nicotiana tabacum* cultivar *‘Xanthi’, Arabidopsis thaliana* Col and *ft-1 Arabidopsis* mutant (Ler ecotype) were used for wild controls.

### Molecular Cloning and Phylogenetic Analysis of FT/TFL1 Homologs

Genomic DNA from eight Rosaceae species was extracted from young leaves as described previously by Wang Z. et al. (2011). Total RNA was extracted according to a previous protocol (Hu et al., 2002). The initial *FT/TFL1* genomic DNA sequences were isolated by homology cloning strategies and genome walking methods (Wang Z. et al., 2011). The degenerated primers were designed according to the *FT/TFL1* sequences from other Rosaceae species. For cloning of the *FT* homologs, the degenerated primers used were: FTF1, 5′-ATGCCTAGGGAHAGGGAYCCYCTTGTT-3′, FTF2, 5′-GCAACAACGGCGGCAAGCTT-3′, and FTR, 5′-CCAGAGCCRCYCTCCCTYTGGCAGTT-3′. For cloning of the *TFL1* homologs, the degenerated primers used were: TFL1F, 5′-TTGGNAGAGTGATAGGAGATGTT-3′, TFL1R, 5′-GAGGAAGGTGKGTTGATTGA-3′. Fusion primer and nested integrated PCR (FPNI-PCR) was used to isolate the unknown sequences flanking the core sequences amplified from the degenerated primers. The full-length *FT/TFL1* cDNA sequence was isolated by specific primers (Supplementary Tables S1–S3). Amino acid sequences were aligned using CLUSTALW MULTIPLE ALIGNMENT with default parameters. Phylogenetic studies were performed using MEGA5 based on the neighbor-joining method (Tamura et al., 2011). Nodal support was estimated by bootstrap analysis and an interior branch test on the basis of 1000 re-samplings.

### Structure Determination

Protein structures of FT and TFL1 homologs were obtained using SWISS-MODEL workspace (Arnold et al., 2006^1^) and visualized by UCSF Chimera (Pettersen et al., 2004). The three-dimensional structures of 3AXY and 1WKO were used as loading template for FT and TFL1, respectively.

### Site-Directed Mutagenesis of Known *FT/TFL1*

The gene splicing overlap extension PCR method (SOE-PCR) (Ho et al., 1989) was used to get a pre-determined point mutagenic site in *FT/TFL1* sequences. We designed a pair of complementary oligo primers in which 1 or 2 base pairs had been altered to introduce a specific mutation into the amplified gene sequence. These mismatch primers mutants (i.e., RoFTmu1F and RoFTmu1R) were paired with unaltered RoFTR and RoFTF primers, respectively, and were used for PCR to generate two DNA fragments with overlapping ends. The two fragments were combined in a subsequent ‘fusion’ reaction PCR using RoFTF and RoFTR primers (Supplementary Table S4). All point mutagenic sequences were introduced into pMD18-T and then pMOG22 vector (Mogen, Leiden, The Netherlands).

### Plasmid Construction and Plant Transformation

The *RoFT, RoTFL1, FaTFL1, PhFT, and AtFD* genes were amplified by PCR from each RNA with the appropriate specific primers (Supplementary Table S4). The amplified products were cloned into pMD18-T vector (Takara) and sequenced. Then the inserts were subcloned into the modified binary vector pMOG22 containing the cauliflower mosaic virus (CaMV) 35S promoter and the Nos terminator.

For *Arabidopsis* transformation, the constructs in binary vectors were introduced into *Agrobacterium tumefaciens* strain GV3101. Transgenic plants were generated by floral dip method and the T1 transformants were selected on hygromycin plates for 1 week in LD (16-h-light/8-h-dark) and then transferred to soil at 20–24°C under long day condition (16-h-light/8-h-dark).

Tobacco was transformed by *Agrobacterium tumefaciens* strain EHA105 according to previously described method (Ning et al., 2012). All transgenic tobacco plants were kept in the greenhouse under a photoperiod of 12-h-light/ 12-h-dark. Data were collected from at least 20 individuals and evaluated by analysis of variance (one-way ANOVA). Means were compared using Duncan’s multiple range test.

### qRT-PCR Analysis

For real time qRT-PCR analysis, samples were harvested from the shoot apex of 45-day-old seedlings of T1 transgenic tobacco plants or 3-week-old seedlings of transgenic *Arabidopsis* plants. Three biological replications were performed randomly for each transgenic line. Total RNA was isolated using Trizol reagent (Takara) according to the manufacturer’s instructions. The first strand of cDNA was synthesized using 2 μg of total RNA as a template with the TransScript^TM^ one-step gDNA Removal and cDNA Synthesis Supermix (Transgen, Beijing, China). The qRT-PCR was performed on 7500 Fast Real-Time PCR System (Applied Biosystems) with SYBR Premix EX Tag^TM^ (Takara). The tobacco *NtEF1α* and *Arabidopsis AtEF1α* transcript were used as an internal standard to calculate the relative expression by the comparative CT (△△CT) method, respectively. The primers for RT-PCR and qRT-PCR are detailed in Supplementary Tables S5, S6.

### Yeast Two-Hybrid Analysis

The coding sequences of *AtTFL1, RoFT, RoFTmu1*/*2*/*3*/*4*/*5, FaTFL1, RoTFL1*, and *PhFT* (all containing the EcoR1 and Sal1 restriction sites at the 5′ and 3′ ends, respectively) were cloned into bait plasmid PGBKT7. *Arabidopsis FT (AtFT)* was also introduced to the PGBKT7 plasmid, using the Nde1 and Sal1 restriction sites, as a positive control. The full-length *Arabidopsis FD* coding sequence *(AtFD)* was cloned into prey plasmid PGADT7 using the Nde1 and BamH1 restriction sites. Yeast cells were transformed using Frozen-EZ Yeast Transformation II^TM^ kit (ZYMO RESEARCH, USA). Co-transformed yeast cells were selected on SD-Leu/-Trp plates. Interactions were tested on SD-Leu/-Trp/-His/-Ade/X-a-Gal selective media. Three independent clones for each transformation were tested.

### Bimolecular Fluorescent Complementation (BiFC) Analysis

Strain of *Agrobacterium tumefaciens* GV3101 carrying the BiFC constructs were used for the infiltration of 5–6-week-old *N. benthamiana* leaves, according to the protocol described by Li et al. (2015). Of which, the coding sequences of *AtFT, AtTFL1, RoFT, RoFTmu1*/*2*/*3*/*4*/*5, FaTFL1, RoTFL1*, and *PhFT* were introduced into the vector pFGC-YC155, respectively. The At*FD* coding sequence was cloned into the vector pFGC-YN173. All vectors were constructed by Gibson assembly method (Gibson et al., 2009). The primers are detailed in Supplementary Table S7. YFP fluorescence was visualized by confocal laser scanning microscope (LSM510 Meta, Zeiss, Germany).

### Expression and Purification of His-Tagged FT Protein

The coding sequences of *AtFT, RoFT, RoFTmu2/3/4/5, PhFT, AtTFL1, RoTFL1*, and *FaTFL1* were amplified with the primers which were used to construct PGBKT7 vectors before (Supplementary Table S7), and finally cloned into the EcoR1/Sal1 (Sac1/Sal1 for *AtFT*) sites of PET-32a vector (NOVAGEN) to obtain PET32a-His-FT. The 10 PET32a-His-FT plasmids were transformed into competent *Escherichia coli Rosetta (DE3)* cells (Transgen, Beijing, China). Fusion protein expression was induced at an OD_600_ of about 0.5 by adding IPTG (isopropyl β-D-1-thiogalactopyranoside) (0.2 mM final concentration), in which the cells were grown overnight and the temperature was shifted from 37 to 16°C. The expressed soluble proteins were purified with Ni-Agarose (CWBIO, Beijing, China) according to the manufacturer’s instructions.

### Fat Western Blotting

18:1-PC (1, 2-Dioleoyl-sn-Glycero-3-phosphatidylcholine) standards was purchased from Larodan (Sweden). The reaction was performed according to the modified protocol described by Stevenson et al. (1998). Of which, a goat anti-rabbit IgG conjugated to alkaline phosphatase (CWBIO, Beijing, China) against 6X histidine was diluted at a 1:10000 level, and the protein bound to the lipid spot was detected by alkaline phosphatase substrate according to the manufacturer’s instructions (Promega).

### Accession Numbers

Sequence data from this article can be found in NCBI under the following accession numbers: *Arabidopsis* AtFT (AF152096); *Beta* BvFT1 (HM448910); *Beta* BvFT2 (HM448912); *Citrus* CiFT (AB027456); *Fragaria* FaFT (CBY25183); *Malus* MdFT*1* (BAD08340); *Malus* MdFT2 (ADP69290); *Nicotiana* NtFT1 (JX679067); *Nicotiana* NtFT2 (JX679068); *Nicotiana* NtFT3 (JX679069); *Nicotiana* NtFT4 (JX679070); *Oncidium* OnFT (ACC59806); *Oryza* Hd3a (AB052944); *Petunia* PhFT (ADF42571); *Photinia* PsFT (AEO72028); *Platanus* PaFT (ACX34055); *Populus* PnFT1 (AB106111); *Populus* PnFT2 (AB109804); *Populus* PnFT3 (AB110612); *Prunus mume* PmFT (CBY25181); *Prunus persica* PpFT (AEO72030); *Pyracantha* PfFT (AEO72029); *Pyrus pyrifolia* PpFT (KF240775); *Rosa* RoFT (CBY25182); *Spiraea* ScFT (AEO72031); *Vitis* VvFT (ABF56526); *Zea* ZmFT (ABW96237); *Arabidopsis* TFL1 (U77674); *Antirrhinum* CEN (CAC21564); *Citrus* CiTFL1 (AY344245); *Fragaria* FaTFL1 (AEO72027); *Malus* MdTFL1-1 (AB162040); *Malus* MdTFL1-2 (AB366643); *Oryza* FDR1 (AF159883); *Oryza* FDR2 (AF159882); *Photinia* PsTFL1 (AEO72024); *Populus* PnTFL1 (AB181183); *Prunus mume* PmTFL1 (AEO72021); *Prunus persica* PpTFL1 (ADL62867); *Prunus yedoensis* PyTFL1 (AEO72023); *Pyracantha* PfTFL1 (AEO72026); *Pyrus pyrifolia* PpTFL1-1 (BAD10962); *Pyrus pyrifolia* PpTFL1-2 (BAK74839); *Rosa* RoTFL1 (AEO72022); *Spiraea* ScTFL1 (AEO72025); *Vitis* VvTFL1 (AF378127); *Zea* ZmTFL1 (ABI98712).

## Results

### *FT/TFL1* Similarity Analysis in Rosaceae Species

**FIGURE 1:**
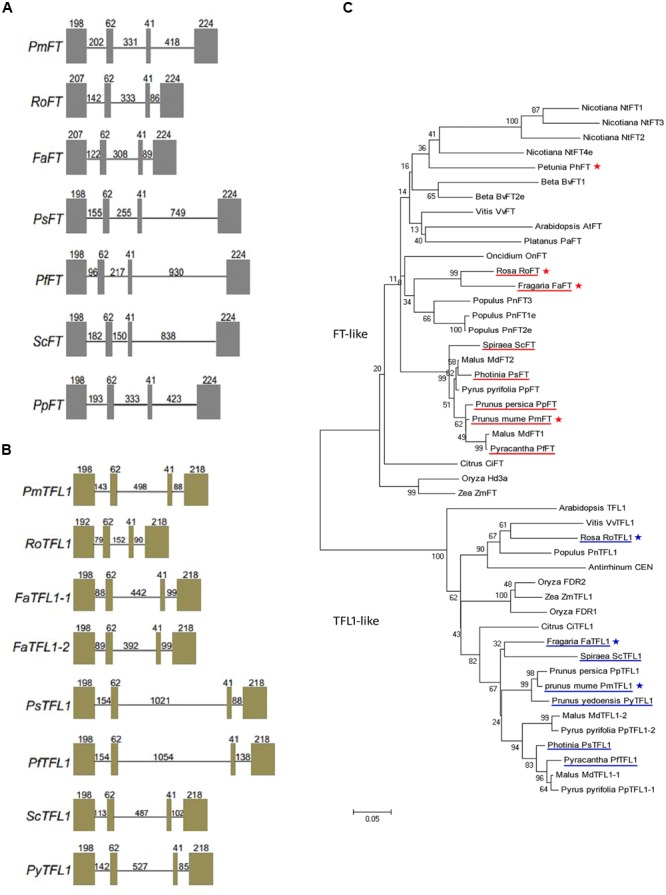
**Gene structures and phylogenetic analysis of the *FT/TFL1* homologs.** Gene structures of: **(A)** *FT* and **(B)** *TFL1* homologs isolated from eight Rosaceae species including *Prunus mume* (*PmFT*), *Rosa* (*RoFT*), *Fragaria* (*FaFT*), *Photinia (PsFT), Pyracantha (PfFT), Spiraea (ScFT), Prunus persica (PpFT); Prunus mume (PmTFL1), Rosa (RoTFL1), Fragaria (FaTFL1), Photinia (PsTFL1), Pyracantha (PfTFL1), Spiraea (ScTFL1), Prunus yedoensis (PyTFL1)*. Boxes indicate exons and lines indicate introns; the numbers represent their corresponding lengths (bp). **(C)** Phylogenetic analysis of the *FT/TFL1* homologs from different plant species. Under-lined genes represent *FT/TFL1* homologs isolated from Rosaceae species and asterisks represent gene sequences used for function identification in this study.

*FT/TFL1* orthologs of eight Rosaceae species, namely, *Spiraea cantoniensis, Pyracantha fortuneana, Photinia serrulata, Fragaria ananassa, Rosa hybrida, Prunus mume, Prunus persica* (only for *FT*) and *Prunus yedoensis* (only for *TFL1*), were isolated. Two *TFL1* copies were isolated from *Fragaria ananassa* genomic DNA, but only one gene copy was isolated from all other genotypes. Each of the isolated *FT/TFL1* sequences contained four exons and three introns. In all isolated genes, the sizes of the second and third exons were the same, i.e., 62 and 41 bp, respectively (**Figures 1A,B**). The seven *FT/TFL1* sequences share 92.09 and 90.59% identity, respectively (Supplementary Figure S1). All *FT/TFL1* homologs from the eight Rosaceae species were found to contain the (putative) crucial amino acid residues of Tyr-85 (for FT) and His-88 (for TFL1). Based on the construction of the phylogenetic tree, it was deduced that all seven *FT* orthologs were clustered within the *FT*-like group and all seven *TFL1* orthologs were clustered within the *TFL1-*like group (**Figure 1C**).

### Functional Determination of the *FT/TFL1* Homologs of Rosaceae Species

For functional study of *FT/TFL1* homologs from eight Rosaceae species, we constructed over-expression vectors harboring *FT* and *TFL1* homologs (cDNA) of *Prunus mume, Rosa hybrida*, and *Fragaria ananassa*. The three species represent different vegetative growth and flowering habit. Two *TFL1* copies were isolated from *Fragaria ananassa* genomic DNA, namely, *FaTFL1-1* and *FaTFL1-2*. There are three single-base differences between the two predicted CDS regions. But only one copy was amplified from the cDNA which shared the same sequence with the predicted CDS region of *FaTFL1-1* gDNA sequence.

**FIGURE 2:**
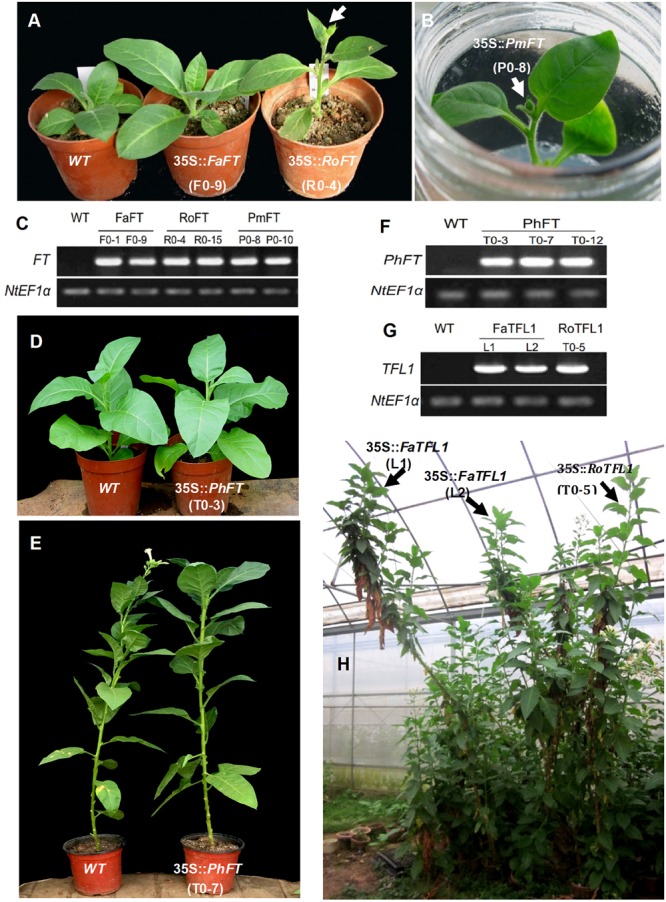
**Phenotypic analysis of transgenic tobacco plants harboring different *FT/TFL1* homologs from various species. (A)** From left to right are wild-type, and transgenic plants harboring *FaFT* and *RoFT*, respectively, after growth for 1 month. **(B)** Tobacco plant harboring *35S::PmFT* and showing visible flower bud in culture box. **(C)** RT-PCR analysis to confirm the *FT* transgenic lines. **(D,E)** Transgenic tobacco plants harboring *PhFT* showing normal growth and no early flowering phenotype after growth for 1.5 and 5 months, respectively. **(F)** RT-PCR analysis to confirm *PhFT* transgenic lines. **(G)** RT-PCR analysis to confirm *FaTFL1* and *RoTFL1* transgenic lines. **(H)** Transgenic tobacco plants harboring *FaTFL1* and *RoTFL1* after growth for 13 months.

According to the results from 20 independent transgenic tobacco lines, the majority of over-expressing *RoFT* and *PmFT* tobacco lines (**Figures 2A–C**), exhibited strongly advanced flowering traits, this was consistent with an earlier preliminary analysis (Ning et al., 2012). At time of flowering, the wild-type had generated 28.6 ± 1.1 leaves, compared with 6.8 ± 1.0 and 5.9 ± 1.1 leaves in the *35S::RoFT* lines R0-4 and R0-15, respectively (**Table 1**). In contrast to the strongly advanced flowering of *RoFT* and *PmFT* lines, the over-expression of *FaFT* in line F0-1 produced a moderately late flowering time (almost 30 days later relative to wild-type). The number of leaves and height remained comparable to the wild-type (**Table 1**). One of the transgenic line F0-9’s flowering time was approximately 50-days later than the wild-type. Thus, there was clearly some functional divergence with respect to the control of flowering between the *FT* orthologs from the different plant species.

**Table 1:** Flowering phenotypes of representative T_1_ transgenic tobacco lines harboring various *FT/TFL*1 homologs.

Genotype	Line label	n	No. leaves on main stem at flowering	Plant height at first flower bud (cm)	Time from seed to first flower bud (days)
*Wt*	*Wt*	10	28.6 ± 1.1e	120.2 ± 3.8f	168.8 ± 6.6h
*35S::RoFT*	R0-4	20	6.8 ± 1.0f	16.4 ± 2.7g	46.9 ± 4.3ij
	R0-15	20	5.9 ± 1.1f	13.4 ± 2.6gh	41.3 ± 2.6j
*35S::PmFT*	P0-8	20	5.5 ± 0.9f	10.6 ± 2.8h	42.5 ± 4.9ij
	P0-10	20	6.4 ± 1.1f	11.5 ± 3.0gh	49.0 ± 2.1i
*35S::FaFT*	F0-1	20	30.5 ± 1.9e	123.4 ± 4.9f	194.6 ± 6.9g
	F0-9	20	38.9 ± 3.1d	130.9 ± 4.4e	218.5 ± 7.8f
*35S::PhFT*	T0-3	20	69.3 ± 3.8c	174.2 ± 4.4cd	294.0 ± 7.1e
	T0-7	20	77.2 ± 2.9b	180.2 ± 3.5b	320.2 ± 6.8c
*35S::RoTFL1*	T0-5	20	66.3 ± 3.6c	172.9 ± 4.5d	291.4 ± 10.2e
	T0-8	20	85.1 ± 3.4a	176.6 ± 4.2c	372.8 ± 14.3b
*35S::PmTFL1*	T0-2	20	68.3 ± 3.1c	173.9 ± 3.2cd	301.0 ± 9.1d
	T0-7	20	79.8 ± 7.3b	185.0 ± 6.5a	385.1 ± 7.9a
*35S::FaTFL1*	L1	20	65.8 ± 3.3c	174.5 ± 5.0cd	294.9 ± 7.0e
	L2	20	80.1 ± 5.1b	187.1 ± 9.8a	387.9 ± 9.2a

*n = number of independent plants analyzed. Values are mean ± SE. Figures followed by common letters within the same column are not significantly different at *P* = 0.05.*

The majority of 35S::*PmTFL1*, 35S::*RoTFL1*, and 35S::*FaTFL1* transformants flowered much later than wild-type plants. Most transformants did not flower in less than 7 months after sowing, as compared to approximately 5.5 months seen in wild-type plants. In some extreme cases, flowering in transformed plants was delayed to over 12 months after sowing (**Figures 2G,H**). As shown in **Table 1**, the two selected lines transformed with 35S::*FaTFL1* had produced as many as over twice leaves on the main stem to wild-type plants by the time of flower initiation. Transformants expressing 35S::*PmTFL1* and 35S::*RoTFL1* showed very similar results to those shown for 35S::*FaTFL1* transgenic lines. Therefore, tobacco plants overexpressing the three *TFL1* orthologs from *Prunus mume, Rosa hybrida*, and *Fragaria ananassa* had an extended vegetative phase and a strongly delayed transition to the reproductive phase.

A similar phenotype to this late flowering imposed by Rosaceae *TFL1* homologes also resulted from the over-expression of a *FT* homolog which was isolated from *Petunia hybrida* (**Figures 2D–F**). The *PhFT* gene contained the Tyr-85 residue and LYN/IYN triplet motif as typical FT sequences, but a Lys-139 residue replaced the normal amino acid in *FT* (i.e., Gln-140); the corresponding residue in *TFL1* was Asp-144 (Supplementary Figure S2). The resulting *35S::PhFT* transgenic tobacco reached over 2 m in height because of extremely late flowering. Thus, it demonstrated a new role of TFL1 although it was identified as an *FT* homolog in our phylogenetic analysis.

### Identification of Key Amino Acids Regulating the Activity of *FT/TFL1* Homologs

**FIGURE 3:**
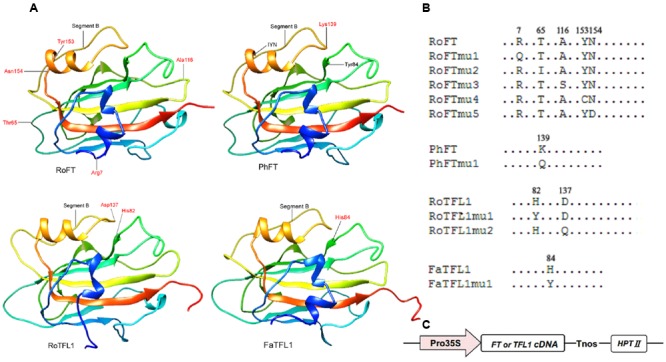
**Crystal structures of FT and TFL1 and maps of point mutated residues. (A)** Cartoon diagrams of four FT or TFL1 homologs. The red high-lighted residues show the corresponding mutated points that were substituted for use in transgenic experiments. The protein pairs: RoFT/PhFT and RoTFL1/FaTFL1 present highly similar crystal structures to each other. **(B)** Diagram mapping the corresponding mutated amino acid residues of FT or TFL1 homologs. **(C)** Schematic map of the T-DNA region (vector pMOG22) used to perform the transgenic experiments.

Since *Rosa FT* (*RoFT*) and *Fragaria FT* (*FaFT*) exhibited quite different effects on flowering time in transgenic tobacco, we compared their sequences in more detail. The two proteins share approximately 88% identity with 13 non-conserved substitutions amongst 20 different amino acids (Supplementary Figures S1, S2), to be key in their flowering time function. We focused on five amino acids, which corresponding to residues 7, 65, 116, 153, and 154 in RoFT. The amino acids at positions 7, 65, 116, and 153 in *RoFT* were changed individually to correlate with the corresponding amino acid residues encoded by *FaFT* (**Figures 3A,B**). In addition, we mutated the amino acid N-154 which is identical between *RoFT* and *FaFT* within the IYN triplet motif of segment C in exon 4. The five resulting mutants were respectively named *RoFTmu1-5* and each was over-expressed under the control of the constitutive CaMV 35S promoter (**Figure 3C**).

**FIGURE 4:**
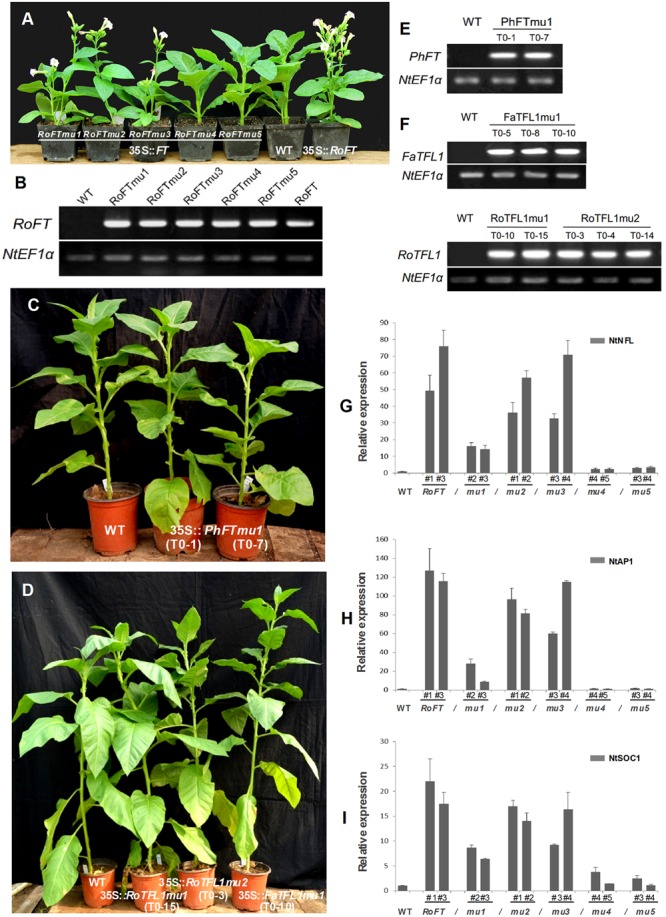
**Phenotypic analysis of transgenic tobacco plants harboring different *FT/TFL1* homologs. (A)** From left to right, 35S::*RoFTmu1-5*, wild-type and 35S::*RoFT* plants, respectively, after growth for 45 days. **(B)** RT-PCR analysis to confirm the transgenic lines. **(C)** From left to right are wild-type, and transgenic plants harboring *PhFTmu1* (two lines) after growth for 3 months. **(D)** From left to right are wild-type, transgenic plants harboring *RoTFL1mu1, RoTFL1mu2*, and *FaTFL1mu1* after growth for 4 months. **(E,F)** RT-PCR analysis to confirm the transgenic lines. **(G–I)** qRT-PCR analysis of endogenous flowering genes in 45-day-old seedlings of transgenic and wild-type tobacco. The transcript levels of: **(G)** *NtNFL*, **(H)** *NtAP1*, and **(I)** *NtSOC1* in different transgenic tobacco lines harboring various point mutations of *FT*. In this analysis, *NtEF1α* was used as a reference transcript. Three biological replications were performed randomly for each transgenic line.

**FIGURE 5:**
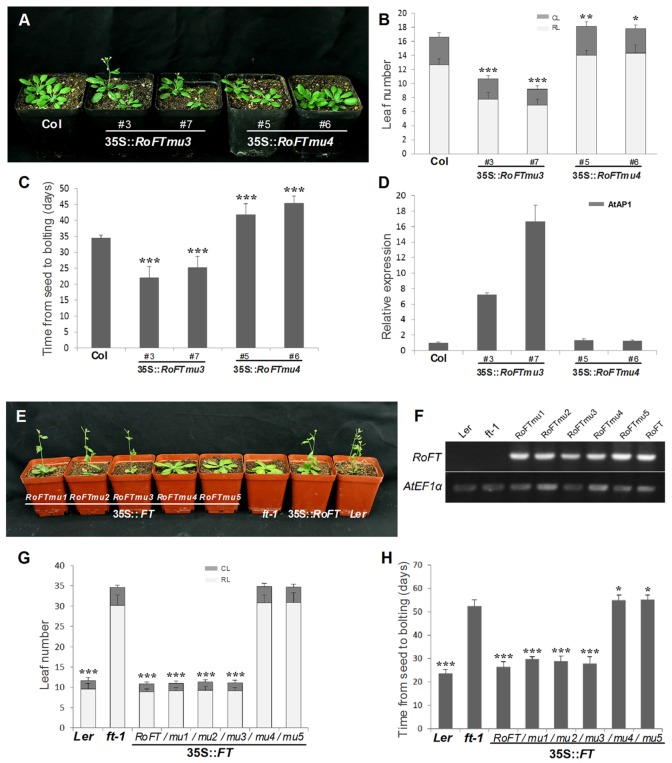
**Phenotypic analysis of ectopically expressing mutated *RoFT* transcripts in the Col and *ft-1* Background. (A)** 25-day-old 35S::*Roftmu3* (A116S) plant (center) flowering 20 days after germination which was earlier than wild-type Col (left) and 35S::*Roftmu4* (Y153C) (right). Leaf number **(B)** and time from seed to bolting **(C)** of wild-type Col and transgenic *Arabidopsis* plants under LD (16-h-light/8-h-dark) conditions. RL, rosette leaves; CL, cauline leaves. **(D)** qRT-PCR analysis of endogenous flowering genes *AtAP1* in 3-week-old seedlings of wild-type Col and transgenic *Arabidopsis* plants. *AtEF1α* was used as a reference transcript. Three biological replications were performed randomly for each transgenic line. **(E)** From left to right, 35S::*RoFTmu1-5, ft-1*, 35S::*RoFT* and *Ler*. 35S::*RoFT* and 35S::*RoFTmu1-3* plants flowering 25 days after germination which were earlier than *ft-1* mutant. **(F)** RT-PCR analysis to confirm the transgenic lines. Leaf number **(G)** and time from seed to bolting **(H)** of *ft-1* and transgenic *Arabidopsis* plants under LD (16-h-light/8-h-dark) conditions. Asterisks show that the values are significantly different between the transgenic lines and the control (^∗^*P* < 0.05; ^∗∗^*P* < 0.01; ^∗∗∗^*P* < 0.001).

Tobacco plants over-expressing *RoFTmu1* (R7Q), *RoFTmu2* (T65I), and *RoFTmu3* (A116S) displayed an early-flowering phenotype, comparable to the native *RoFT* in transgenic tobacco. In contrast, *35S::RoFTmu4* (Y153C) and *35S::RoFTmu5* (N154D) transgenic plants showed a strong late flowering phenotype (**Figures 4A,B**). As shown in **Table 2**, 35S::*RoFTmu1*, 35S::*RoFTmu2*, and 35S::*RoFTmu3* tobacco plants flowered after producing approximately 8–10 leaves over 2 months of growth. By contrast, the majority of the 35S::*RoFTmu4* and 35S::*RoFTmu5* transformants had a much delayed flowering time, requiring 210 ± 27.1 and 248.1 ± 32.7 days of growth, respectively. We also ectopically expressed 35S::*Roftmu3* (A116S) and 35S::*RoFTmu4* (Y153C) in *Arabidopsis* Col. 35S::*RoFTmu3* (A116S) plants showed a marked early flowering phenotype, with approximately 50% the number of leaves as found in the wild-type Col at floral initiation (**Figures 5A–C**). Transgenic 35S::*Roftmu4* (Y153C) *Arabidopsis* flowered slightly later than the corresponding wild-type Col (**Figures 5A–C**). In addition, overexpressing *RoFTmu1, RoFTmu2*, and *RoFTmu3* within *ft-1* mutant (*Ler* ecotype) resulted in significant early flowering compared to *ft-1* plants (**Figures 5E,F**). As shown in **Figure 5G**, *ft-1* mutant harboring *35S::RoFTmu3* possessed 9.1 ± 0.9 rosette leaves at the time of bolting, which is almost consistent to that resulted from 35S::*RoFT* (8.9 ± 0.7), while, *ft-1* mutant had produced as many as >3-fold leaves (30.2 ± 2.5) until flowering. Meanwhile, the flowering time was much earlier than those *ft-1* plants (**Figure 5H**).

**Table 2:** Flowering phenotypes of regenerated T_0_ transgenic tobacco lines harboring mutated *RoFT* transcripts.

Genotype	n	No. leaves on main stem at flowering	Plant height at first flower bud (cm)	Time between transformed plantlet regeneration and first flower bud (days)
*Wt*	6	26.7 ± 1.0c	121.7 ± 4.4d	160.7 ± 6.6c
*35S::RoFT*	20	8.3 ± 0.9d	18.7 ± 1.9e	47.6 ± 6.3d
*35S::RoFTmu1*	22	9.3 ± 1.1d	18.6 ± 1.8e	57.8 ± 8.9d
*35S::RoFTmu2*	24	9.3 ± 0.8d	20.3 ± 2.1e	53.9 ± 8.8d
*35S::RoFTmu3*	22	9.0 ± 1.2d	20.0 ± 2.1e	47.3 ± 9.6d
*35S::RoFTmu4*	5	26.4 ± 0.5c	126.0 ± 4.2cd	152.0 ± 10.4c
	15	41.7 ± 10.3b	148.1 ± 11.8b	210.7 ± 27.1b
*35S::RoFTmu5*	3	27.3 ± 0.6c	129.0 ± 3.6c	161.7 ± 7.6c
	17	49.3 ± 10.3a	161.1 ± 13.0a	248.1 ± 32.7a

*n = number of independent plants analyzed. Other codes are the same as given in **Table 1**.*

It has been reported that the opposite roles of FT and TFL1 are related to the conserved amino acids His-88 and Asp-144 in TFL1 (Hanzawa et al., 2005; Ahn et al., 2006). To examine whether these amino acids is also conserved in other plant species, we constructed mutants *RoTFL1mu1* (H82Y), *RoTFL1mu2* (D137Q), *FaTFL1mu1* (H84Y), and *PhFTmu1* (K139Q) (**Figure 3B**), and transferred them into tobacco plants. As shown in **Figures 4C–F**, no early flowering phenotype was observed in any of these transformants, as compared to wild-type tobacco. In fact, some of these transgenic plants remained in the vegetative growth phase for over 11 months (**Table 3**).

**Table 3:** Flowering phenotypes of regenerated T_0_ transgenic tobacco lines harboring mutated *TFL1*-like transcripts.

Genotype	n	No. leaves on main stem at flowering	Plant height at first flower bud (cm)	Time between transformed plantlet regeneration and first flower bud (days)
*Wt*	5	28.8 ± 1.3b	125.8 ± 4.1b	163.4 ± 4.8b
*35S::RoTFL1mu1*	2	25.5 ± 0.7b	116.5 ± 2.1b	131.5 ± 4.9b
	19	64.7 ± 10.1a	170.5 ± 11.0a	287.9 ± 33.1a
*35S::RoTFL1mu2*	2	29.5 ± 0.7b	129.0 ± 1.4b	169.0 ± 1.4b
	19	64.1 ± 12.0a	169.4 ± 10.4a	284.5 ± 34.6a
*35S::FaTFL1mu1*	4	23.8 ± 0.5b	119.3 ± 1.0b	127.5 ± 2.9b
	16	60.6 ± 11.4a	164.8 ± 10.2a	269.4 ± 31.5a
*35S::PhFTmu1*	20	63.2 ± 7.8a	172.1 ± 9.2a	286.5 ± 27.5a

*Codes are the same as given in **Tables 1, 2**.*

### Expression of Floral Genes in Specific Transgenic Plants

According to previous studies (Abe et al., 2005; Wigge et al., 2005; Searle et al., 2006), the FT protein activates the floral meristem identity genes *APETALA1* (*AP1*), *SOC1*, and *LFY*. These have been identified as important floral pathway integrators in *Arabidopsi*s. The expression of the *LFY, AP1*, and *SOC1* orthologs, *NtNFL, NtAP1*, and *NtSOC1* of tobacco was evaluated by real-time RT-PCR in the shoot apex of 45-day-old seedlings of T1 transgenic lines and wild-type (Smykal et al., 2007; Zhang et al., 2014). *NtNFL* (**Figure 4G**), *NtAP1* (**Figure 4H**) and *NtSOC1* (**Figure 4I**) were highly up-regulated in 35S::*RoFT*, 35S::*RoFTmu1*, 35S::*RoFTmu2*, and 35S::*RoFTmu3* transgenic tobacco plants, which all showed an early-flowering phenotype. There was no obvious change in transcript levels of these endogenous genes in the 35S::*RoFTmu4* and 35S::*RoFTmu5* transgenic plants, which showed a late-flowering phenotype. Similarly, the expression of *AtAP1*, one of a downstream gene of FT, was up-regulated in 35S::*RoFTmu3* transgenic *Arabidopsis* plant (**Figure 5D**).

### Interaction of AtFD with *FT/TFL1* Homologs

**FIGURE 6:**
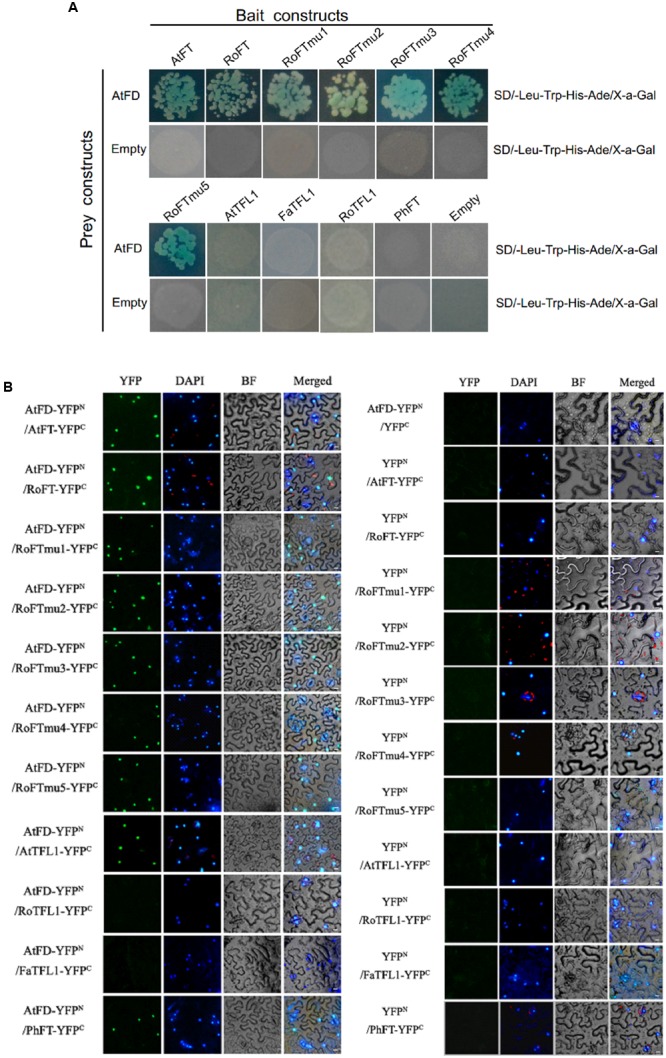
**Interaction of FT/TFL1 and AtFD proteins. (A)** Yeast two-hybrid analysis to study the interaction among different FT/TFL1 homologs. Transformed yeast cells (10^3^ or 10^4^ diluted) were grown on selection medium containing X-a-Gal. **(B)** BiFC analysis of protein interactions between different FT/TFL1 homologs and AtFD in *N. benthamiana* leaf epidermis cells. YFP, YFP fluorescence; DAPI, DAPI fluorescence; BF, blight field image; Merged, merge of YFP, DAPI, and BF. The AtFT with AtFD interaction was used as a positive control. Bars = 10 μm.

According to the literature, both FT and TFL1 can interact with the bZIP transcription factor FD, which regulates the expression of several flower meristem (FM) identity genes (Abe et al., 2005; Benlloch et al., 2011). In order to examine whether Rosaceae FT/TFL1 homologs are able to interact with FD, and whether single amino acid substitutions in RoFT can affect the interaction, we performed yeast two-hybrid assays. *Arabidopsis* FD (AtFD) was used as a prey, and various FT/TFL1 homologs were cloned as the bait. Transformed yeast cells growing on SD/-Leu-Trp selection medium were shown in Supplementary Figure S3. The results indicated that in yeast, AtFD was able to interact with AtFT, RoFT and five RoFTmu1-5 point-mutated forms. However, no interaction was observed of AtFD with AtTFL1, FaTFL1, RoTFL1, or PhFT (**Figure 6A**). To further verify the interaction of FT/TFL1 homologs and AtFD, the N-terminal half of YFP fused to AtFD (AtFD-YFP^N^) and the C-terminal half of YFP fused to FT (FT-YFP^C^) were employed to perform BiFC test. YFP fluorescence was obviously observed in the nucleus (**Figure 6B**). The two results indicated that, except FaTFL1 and RoTFL1, the other FT/TFL1 homologs were able to interact with AtFD in the nucleus.

### PC Binding Activities *In Vitro*

**FIGURE 7:**
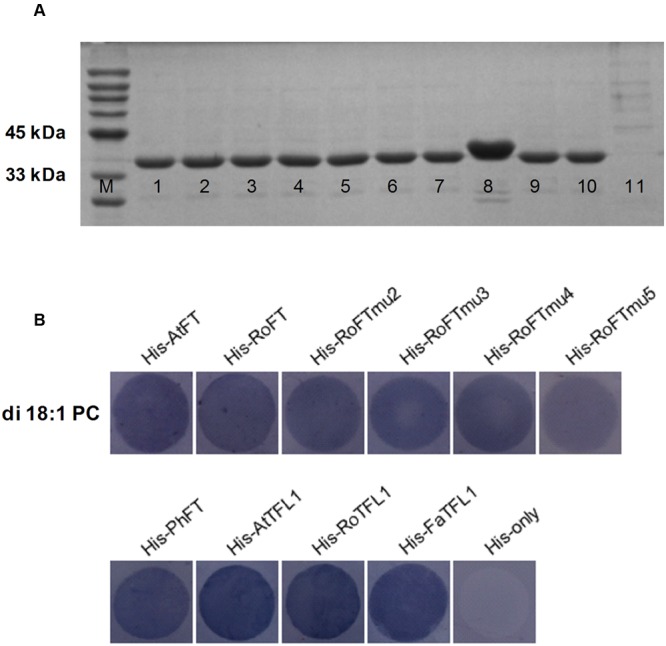
**FT proteins binding to phosphatidylcholine (PC). (A)** His-FT/TFL1 purified proteins on SDS-PAGE Gel. M, Protein Marker; 1-11, His-AtFT, His-RoFT, His-RoFTmu2, His-RoFTmu3, His-RoFTmu4, His-RoFTmu5, His-PhFT, His-AtTFL1, His-RoTFL1, His-FaTFL1, His-only. **(B)** Various His-FT/TFL1 proteins binding to di 18:1 PC on the membrane. The His-AtFT and His-only with PC binding was used as a positive and negative control, respectively.

To test whether RoFT, point mutated RoFT and PhFT have the lipid-binding property, we performed a Fat-Western blotting using membrane-lipid overlay assays. All of the AtFT, RoFT, RoFTmu2/3/4/5 PhFT, AtTFL1, RoTFL1 and FaTFL1, with a C-terminal histidine tag, were expressed and purified (**Figure 7A**). The fusion proteins were hybridized with PC-spotted nitrocellulose membrane and detected using anti-His antibodies respectively. A clear binding of His-FT/TFL1 to PC was detected (**Figure 7B**) though these FT/TFL1 proteins have, not have or even in verse roles in flowering modulating.

## Discussion

### *FT/TFL1* Homologs Exhibit Both Functional Similarity and Diversity across Various Species

The plant PEBP family can be divided into three major clades, i.e., the *FT-*like, *MFT-*like, and *TFL1-*like clades. The first two act as promoters of flowering, whereas *TFL1-*like clade acts as strong repressors of the response. Within the eight Rosaceae species, the *FT/TFL1* homologs show high sequence identity (Supplementary Figure S1). Ectopic expression of *PmTFL1, RoTFL1*, and *FaTFL1* in tobacco extended the vegetative phase and resulted in a significant delay in flowering. It is indicated that *TFL1* homologs play a conservative role in controlling flowering time as previously reported for *AtTFL1*. However, most tobacco overexpressing *PmFT* and *RoFT*, displayed extremely advanced flowering. Contrarily, overexpression of *FaFT* did not promote flowering but, instead, caused slightly delayed by 1–2 months than the wild-type (**Figures 2A,B** and **Table 1**). The results demonstrated a divergence role of *FT* homologs between different species.

### *FT* Homologs Naturally Evolved to Have Diverse Roles in Flowering Time Control

It has been reported that AtFT and AtTFL1 may demonstrate interchangeable roles by replacing a single amino acid (Hanzawa et al., 2005; Hou and Yang, 2009) or a larger protein segment (Ahn et al., 2006; Pin et al., 2010). Tyr-85 in AtFT and His-88 in AtTFL1 have been identified as two key residues that determine the respective FT and TFL1 functions (Hanzawa et al., 2005). It is interesting that Tyr-85 and His-88 are conserved in all FT and TFL1 proteins from the eight Rosaceae species, respectively (Supplementary Figure S1). Sequence comparison analyses showed that there are only 13 non-conserved substitutions between *Rosa* FT (RoFT) and *Fragaria* FT (FaFT), but nevertheless the two genes demonstrated opposite functions in controlling flowering time in transgenic plants (**Figure 2A**). In *Arabidopsis*, protein segment B, in conjunction with the adjacent segment C, has been implicated as essential for *FT-*like activity (Ahn et al., 2006). However, within this segment B we found only one residue is different between RoFT and FaFT, i.e., Glu-139 in RoFT compared to Gly-139 in FaFT and other FT homologs (Supplementary Figure S2). Thus, we suggest that protein segment B is not critical to the activity of FaFT as a flowering repressor. Previous study showed that FT protein is transported from the leaves, where it is synthesized, to the shoot apex where it then interacts with FD, and so leads to the activation of floral meristem identity genes *AP1, LFY*, and *SOC1* (Abe et al., 2005; Wigge et al., 2005; Searle et al., 2006). The expression of the endogenous genes *NtNFL, NtAP1*, and *NtSOC1* were highly up-regulated (49-, 127-, and 22-fold, respectively) in *35S::RoFT* transgenic tobacco line #1 (**Figures 4G–I**). The three site-directed mutants RoFTmu1-3 acted as promoters of flowering in transgenic tobacco lines and *ft-1* plants (**Figures 4A, 5E**), and resulted in the elevated expression of the endogenous genes, the same as seen in response to RoFT. By contrast, RoFTmu4 and RoFTmu5 demonstrated TFL1-like function in the flowering time, and the expression of *NtNFL, NtAP1* and *NtSOC1* in tobacco transformed with these constructs was about twofold higher than that of the control (**Figures 4G–I**). While we cannot rule out complexities that might arise from co-suppression in specific constructs, considering the consistent phenotypes between different ectopic transformants, it suggests that the phenotypes were due to the over-expression of different site-mutated RoFT.

### Tyr-85 and Gln-140 Amino Acids Are Not Sufficient for the Promotion of Flowering by *FT* Homologs

*PhFT* from *Petunia hybrida* shares 71.0 and 54.4% identity with *AtFT* and *AtTFL1*, respectively, and it encodes a typical FT residue Tyr-85 and an important IYN triplet motif located in segment C. However, Lys-139 of PhFT differs from both counterparts from *Arabidopsis* FT (Gln-140) and TFL1 (Asp-144). Phylogenetic analysis placed *PhFT* in a cluster with *FT-*like genes (**Figure 1C**), suggesting a putative FT-like function. Over-expression of *PhFT* in tobacco did not promote early flowering (**Figures 2D,E**) instead, strongly suppressed flowering of the transgenic tobacco. With a mutant *PhFTmu1* (K139Q), ectopic expression of *PhFTmu1* in tobacco was found with late-flowering (**Figure 4C**). These results of transgenic analysis were highly reminiscent of the *FT-*like repressor activity of *BvFT1* in sugar beet (*Beta vulgaris* subsp. *vulgaris*), which exists alongside its antagonistic paralog *BvFT2*. Although both of these *Beta vulgaris* genes encode Tyr-85/Gln-140 residues and the IYN triplet, they demonstrate a naturally evolved antagonistic function (Pin et al., 2010). Similar findings have also been found in the FT gene family of tobacco and *Dimocarpus longan* (Harig et al., 2012; Heller et al., 2014). Thus, the presence of Tyr-85, Gln-140 and triplet IYN residues is not sufficient to indicate whether the FT-like proteins undertake the role of flowering promoter or not. It has been reported that the three differing amino acids in segment B, forming an external loop, are the major cause of the *BvFT1* and *BvFT2* antagonistic function (Pin et al., 2010). However, analysis of the 14-amino-acid segment B of *PhFT* by crystal structure analysis indicated a close resemblance to the tertiary structure of *Arabidopsis* FT. Thus, further investigations are needed to elucidate the real reason why both the PhFT and PhFTmu1 proteins did not function to promote flowering in tobacco plants, as predicted according to their key sequence traits.

### TFL1 Substitution with Key Amino Acids from FT Did Not Promote Flowering in Transgenic Tobacco

Previous reports described transgenic plants expressing the site-directed mutant *TFL1* genes 35S*::AtTFL1*-H88Y (Hanzawa et al., 2005) and 35S*::OnTFL1*-H85Y (Hou and Yang, 2009) to show an early flowering phenotype, similar to that of *Arabidopsis* plants overexpressing native *FT*. Here, we have described transgenic tobacco plants over-expressing *Rosa TFL1* (*RoTFL1*) and *Fragaria TFL1* (*FaTFL1*) to show a late-flowering phenotype (**Figure 2H**). Specific mutations were introduced into these Rosaceae genes, corresponding to the putative key functional His-88 and Asp-144 residues of AtTFL1. However, these mutated genes did not result in early-flowering phenotypes in the transgenic plants (**Table 3**), which is thereby inconsistent with previous report. Based on our study in transgenic tobacco, key amino substitution is not sufficient to promote flowering via *RoTFL1* and *FaTFL1* (**Figure 4D**).

### Site-Directed Mutations of IYN Triplet Motif Resulted in Loss of FT Function

According to a previous report (Ahn et al., 2006), exon 4 of *Arabidopsis* FT plays a critical role in determining FT/TFL1 function. The exon 4 sequence contains four segments, A–D, and segments B and C are necessary for FT-like activity. These segments are also found in the TFL1 protein but, whereas the B and C sequences are highly conserved in many FT orthologs, they appear to have diverged in proteins with TFL1-like activity (Ahn et al., 2006). In the segment B encoded by *RoFT*, a single residue (Glu-139) is different from other *FT* homologs (Supplementary Figure S2). Thus, considering that the consensus sequence of *FT* orthologs contains a Gly residue at this corresponding site in the B segment and, despite this, *RoFT* still functions as a flowering promoter, we suggest that the contrary action of the *FaFT* gene-product as a floral repressor does not hinge on the sequence of segment B in exon 4. Among our five *RoFT* mutants, three mutants outside of IYN triplet led to an early flowering phenotype, similar to that mediated by over-expression of the unaltered *RoFT* gene. By contrast, two mutants within the IYN triplet motif of segment C, were not effective in the promotion of flowering and even to some extent, appeared to act similarly to a *TFL1*-like floral repressor (**Figures 4A, 5A,E**).

### Interaction of FT Homologs with FD Protein and PC-Binding Ability is Independent to Promote Flowering

Using yeast two-hybrid assays, Jang et al. (2009) reported that *Arabidopsis* FT, but not TFL1, interacted with FD. However, Hanano and Goto (2011) used the BiFC technique to demonstrate that both TFL1 and FT can interact with FD within the plant cell nucleus (Hanano and Goto, 2011). In our yeast two-hybrid assays, FaTFL1 was found not to interact with FD, consistent with the findings of Jang et al. (2009) but different with Abe et al. (2005). However, we also found that PhFT, in spite of having high sequence similarity to FT, showed the same interaction pattern as FaTFL1. Our system was able to verify that native *Arabidopsis* FT interacted with FD. RoFT and the five corresponding point mutated protein forms were all shown to interact with AtFD in a similar way to the native *Arabidopsis* FT, which is also strongly supported by our BiFC system (**Figure 6**). In addition, ectopic overexpression of AtFD led to 2–3-months early-flowering in tobacco (Supplementary Figure S4 and Table S8), which showed that the AtFD is functionally active in tobacco as is the case of 35S::*AtFD* in *Arabidopsis* (Abe et al., 2005; Wigge et al., 2005). Since over-expression of the *RoFTmu4* did not promote flowering in tobacco or *Arabidopsis*, we conclude that the physical interaction of FT homologs with the FD protein is not sufficient to bring about the promotion of flowering. These results also indicate that the substitution of a single amino acid residue of RoFT does not necessarily have a major impact on its interaction with FD but may, nevertheless, change its role in the control of flowering. Other interaction partners specific to FT or TFL1 are likely to exist, and this is supported by other studies (Jang et al., 2009; Taoka et al., 2011; Ho and Weigel, 2014). On the other hand, the diversity of interaction with AtFD in TFL1 homologs, verified by yeast two-hybrid and BiFC system, also show no correlation to their roles in flowering delaying. Though FT/TFL1 share a similar 3D structure with animal PEBP with an anion binding pocket, neither FT nor TFL1 were shown to bind any phospholipids *in vivo*. In another study, point mutation of the *Arabidopsis* FT at Asp71 located in the deep pocket did not affect FT activity (Ho and Weigel, 2014). So the significance of the pocket is unclear.

It has been reported that FT binds the phospholipid phosphatidylcholine (PC), a component of cellular membranes whose higher level accelerates flowering. Two models have been proposed to explain the effect of PC on flowering control (Nakamura et al., 2014). As a component of the nuclear membrane, PC may attract free FT from the cytosol into nucleus to promote flowering. Alternatively, PC-containing vesicles could help trafficking of FT to FD. Our FT-lipid assay result shows that whether they promote flowering or not, all FT/TFL1 homologs have the lipid-binding properties (**Figure 7B**). Thus, it is also deduced that lipid-binding and flowering promotion were two independent events. Considering TFL1 homologs have opposite function in controlling flowering, the PC-binging ability may imply other functions such as in mobile signaling. The *TFL1* gene is transcribed in the central region of the SAM, and the protein spreads throughout the IM (dose not reach FM). By contrast, FT is produced in leaves and then is moved into SAMs (Bernier and Périlleux, 2005; Conti and Bradley, 2007; Wickland and Hanzawa, 2015). TFL1 was reported to play a role in endomembrane trafficking to protein storage vacuoles (PSVs) (Sohn et al., 2007). In addition to the fact that TFL1 protein is located in both the nucleus and cytoplasm, thus, TFL1 maybe shuttle FD from nuclei to PSVs, in nuclei where FT recruits FD, to block FD-dependent transcription occurs (Hanano and Goto, 2011). It also implies the TFL1 functions obviously in protein trafficking to PSVs from that the PC binding of His-TFL1 looks stronger than His-FT.

Collectively, beside description of the functional divergences in many FT/TFL1 homologs, our data have also shown that many novel amino acids change can switch FT-like activity to TFL1-like activity. On the other hand, it is also verified that the divergence of flowering time modulating by FT/TFL1 homologs is independent to its interaction and binding activities.

## Author Contributions

GN and ZW designed the experiments and drafted the manuscript. RY, UD, JM, YZ, JL, and YS participated in the coordination of the experiments. GN, JZ, and MB thoroughly revised the manuscript and finalized the manuscript. All the authors read and approved the manuscript.

## Conflict of Interest Statement

The authors declare that the research was conducted in the absence of any commercial or financial relationships that could be construed as a potential conflict of interest.

## Footnotes

1 http://swissmodel.expasy.org
