# Supplementary material for: The Divergence of Flowering Time Modulated by FT/TFL1 Is Independent to Their Interaction and Binding Activities
Source: Front Plant Sci. 2017 May 8;8:697. doi: 10.3389/fpls.2017.00697 (PMC5421193; doi:10.3389/fpls.2017.00697)
Supplement: Supplementary file 1 [file Table_1.PDF]

Table S1.

Gene specific primers used in FPNI-PCR to generate the complete *FT/TFL1* coding sequences.

Genus	*Name*	*Primer sequence(5'-3')*	*Primer use*
	FTF1	ATGCCTAGGGAHAGGGAYCC YCTTGTT	Degenerate primer
	FTF2	GCAACAACGGCGGCAAGCTT	Degenerate primer
	FTR	CCAGAGCCRCYCTCCCTYTGGCAGTT	Degenerate primer
Prunus mume	PmFT		
	5'-extension	ATTACTATTGGCAGGAATGTAGGAGC	1st PCR primer
		CTCCCCCCAAACCAAGAAAAAAAGC	2nd PCR primer
		GAGGTGAGCCATATTGTATTGGTGG	3rd PCR primer
	3'- extension	TTGATGTAGGGCAAGAGATTGTGTGT	1st PCR primer
		GGGATTCATCGCTTTGTTTTGGTGTT	2nd PCR primer
		AGGCAAACAGTGTATGCTCCAGGGTG	3rd PCR primer
Rosa hybrida	RoFT		
	5'- extension	TGGAGCATATACTGTTTGCCTACCC	1st PCR primer
		TTGCTGGAACTAGAAGTAGCCCGAT	2nd PCR primer
		GAAGTAGCCCGATTCAACAAAGTGGA	3rd PCR primer
	3'- extension	CCAGCAAGCTAATATATAAACGCAC	1st PCR primer
		GGTAGGCAAACAGTATATGCTCCAGA	2nd PCR primer
		CGGTGGCCGCTGTCTATTATAACTG	3rd PCR primer
Fragaria ananassa	FaFT		
	5'- extension	CTCTGGCAGTTACAAAAGACAGCGGC	1st PCR primer
		TCCTGGAGCATACACTGTTTGCCTTC	2nd PCR primer
		GGTGCGTTTACATAGTATCTTGCTGG	3rd PCR primer
	3'- extension	CCAGCAAGATACTATGTAAACGCA	1st PCR primer
		GCAGGCCAAGAGATTTTGAGCTATG	2nd PCR primer
		CTGTCTTTTGTAACTGCCAGAGGGAG	3rd PCR primer
Photinia serrulata	PsFT		
	5'- extension	TATGAGTAATATACGTGGGCTTGCTT	1st PCR primer
		AAAAGTGGAATTCTCCGCATCATCT	2nd PCR primer
		TGCAGTAGAAACAACTCACCGAAGCT	3rd PCR primer
	3'- extension	ATGCAGGGCAAGAGATCGTGTGTTAC	1st PCR primer
		GGATTCATCGCTTTGTTTTTGTGCTG	2nd PCR primer
		CTTCAATACCAAAGACTTCGCCGAGC	3rd PCR primer
Pyracantha fortuneana	PfFT		

	5'- extension	ATTCCTTTAGGTTGGGGTCACTTG	1st PCR primer
		GGATGGATGGAAGATGGAAACTTACC	2nd PCR primer
		TCCTTAGTACCGTAGGTCACCCTCAG	3rd PCR primer
	3'- extension	ACCCTTATATTCAAGCATGAACACT	1st PCR primer
		GGGTAGGCAAACAGTGTATGCTCCG	2nd PCR primer
		TGTCTGTCGTCCATTTTAACTGCC	3rd PCR primer
Spiraea cantoniensis	ScFT		
	5'- extension	GCTGGAATATCCGTCACCAACCTG	1st PCR primer
		ACTTGGACTGGGTGCATCAGGATC	2nd PCR primer
		CAGACAAACATACAAAGAGAGCGTG	3rd PCR primer
	3'- extension	CCAATTCCAAAAGACTTAAAAGGGC	1st PCR primer
		CAAATAACATTCAAGTTTTTCCGAC	2nd PCR primer
		ATATGCTGATGATCGCCCTGTGTA	3rd PCR primer
Prunus persica	PpFT		
	5'- extension	AAGCAAGAGGATGGATTGACCCCGTAC	1st PCR primer
		CCAGAGTGTAGAAAGTCCTAAGATCATC	2nd PCR primer
		TGACCCTGAGAGAAACAGACCTTGT	3rd PCR primer
	3'- extension	TCGATTGGTCTAGTCTACAAAGGC	1st PCR primer
		GTAGGGCAAGAGATTGTGTGTTATG	2nd PCR primer
		GCGCCAGAACTTTAATACTAGAGACTT	3rd PCR primer
	TFL1F1	TTGGNAGAGTGATAGGAGATGTT	Degenerate primer
	TFL1R1	GAGGAAGGTGKGTTGATTGA	Degenerate primer
Prunus mume	PmTFL1		
	5'- extension	ACGGAAGGACTGTTCGGTGCTTGTATC	1st PCR primer
		ACTCAAATTGATCCGATAATGGAGACTC	2nd PCR primer
		GGTGAAACAATCAAGAACATCTCCTATC	3rd PCR primer
	3'- extension	CAATGGTCCAGATACAAGCACCGAACAGT	1st PCR primer
		TGACAGATATTCCAGGCACCACAGATG	2nd PCR primer
		ACAGGTTTGTGTTTGTTCTCTTCAAGCAG	3rd PCR primer
Rosa hybrida	RoTFL1		
	5'- extension	GTTGTTCCTGGGGTATCCGTGACTATCCTG	1st PCR primer
		CATGTTGAATCTGCCCTATTATTGCTGT	2nd PCR primer
		ACCAGTGCAAGTGCTCCTTCAGATATG	3rd PCR primer
	3'- extension	CGGATACCCCAGGAACAACTGACAACAC	1st PCR primer
		GAGGTGGTGAAATATGAAATGCCGAGG	2nd PCR primer
		TTCAAGCAGAAAGGTAGGCAAACAGT	3rd PCR primer
Fragaria ananassa	FaTFL1		
	5'- extension	TAACCTGGACAATGTGTTCGGTCAATAC	1st PCR primer
		GTGCAGGTGCTCTTTCAAATAAGGATC	2nd PCR primer

		GCAGACGAGCTTGCTGTTGTAAGAGAC	3rd PCR primer
	3'- extension	TAGTGATCCTTATTTGAAAGAGCACCTGC	1st PCR primer
		CCGAACACATTGTCCAGGTTAATTTATAG	2nd PCR primer
		GTTCTCCTTTACATGGTCATTCACG	3rd PCR primer
Photinia serrulata	PsTFL1		
	5'- extension	TCACCTGCAAGATTAAGAAAACTATCACAAG	1st PCR primer
		TGAATCTCAACTCTAGGTTTGGCTGTG	2nd PCR primer
		GTCCATTGCAGACTAGCTTGGTGTTG	3rd PCR primer
	3'- extension	TGGTTCAATTTTACACTCCAAGGTCAC	1st PCR primer
		ACGTACTATCATTTCCCAAAGCCCTAT	2nd PCR primer
		CCACAGATGCCACATTTGGTAAGTCCT	3rd PCR primer
Pyracantha fortuneana	PfTFL1		
	5'- extension	ATTCATAGTGGTGGTGTATTCCAGTGC	1st PCR primer
		TTAGATAAGGATCACTAGGGCCAGGA	2nd PCR primer
		ACATCTCATATCCCCTCCTTGAATCT	3rd PCR primer
	3'- extension	CACATTTTCCTGGCCCTAGTGATCCTT	1st PCR primer
		ATTATGGGCCGGAGATTTTACGGTATT	2nd PCR primer
		TCGTTAAGCATCAACCATAGTTACCAG	3rd PCR primer
Spiraea cantoniensis	ScTFL1		
	5'- extension	TTGTTTAGGGTTTTGAGTAGGCGTATCG	1st PCR primer
		TCATGTAAAGTACCAAAGTGAAGAAGGA	2nd PCR primer
		CTGAAAGTGACAGTCATTTTTGTGGTGG	3rd PCR primer
	3'- extension	AGCCGTAGGGTAAAACTGAATTGACTGAC	1st PCR primer
		AATAATATTCAGGACCTACATGTCACGGA	2nd PCR primer
		TGACAGACATTCCAGGCACCACAGA	3rd PCR primer
Prunus yedoensis	PyTFL1		
	5'- extension	TCGCTTAAATAAGGATCACTAGGGGCAG	1st PCR primer
		CGCACAAGAGATGCAAAGTAAAATCAAGT	2nd PCR primer
		AGGTTTGGTGGTGACAGCAGAAGGATAG	3rd PCR primer
	3'- extension	GGTCCAGATACAAGCGCCTAACAGTCCT	1st PCR primer
		AGCAGGACTTCAGTCAAAGCTTCTAATAG	2nd PCR primer
		GTCAGCGTATACCTCTACATGCACCTTG	3rd PCR primer

Table S2.

The FAD primers and universal primers used in FPNI-PCR.

Name	*Primer sequence*	*Primer use*
Mad1	GTAATACGACTCACTATAGGGCACGCGTGGT NTCGA STWTS GWGTT	1st PCR primer
Mad2	GTAATACGACTCACTATAGGGCACGCGTGGT NGTCG ASWGA NAWGAA	1st PCR primer
Mad3	GTAATACGACTCACTATAGGGCACGCGTGGT WGTGN AGWAN CANAGA	1st PCR primer
Mad4	GTAATACGACTCACTATAGGGCACGCGTGGT AGWGN AGWAN CAWAGG	1st PCR primer
Mad5	GTAATACGACTCACTATAGGGCACGCGTGGT NGTAW AASGT NTSCA A	1st PCR primer
Mad6	GTAATACGACTCACTATAGGGCACGCGTGGT NGACG ASWGA NAWGAC	1st PCR primer
Mad7	GTAATACGACTCACTATAGGGCACGCGTGGT NGACG ASWGA NAWGAA	1st PCR primer
Mad8	GTAATACGACTCACTATAGGGCACGCGTGGT GTNCG ASWCA NAWGTT	1st PCR primer
Mad9	GTAATACGACTCACTATAGGGCACGCGTGGT NCAGC TWSCT NTSCTT	1st PCR primer
UnAD1	GTAATACGACTCACTATAGGGC	2nd PCR primer
UnAD2	ACTATAGGGCACGCGTGGT	3rd PCR primer

Table S3.

Gene specific primers used to isolate complete *FT/TFL1* coding sequences.

Genus	*Name*	*Forward primer (5'-3')*	*Reverse primer(5'-3')*
Prunus mume	PmFT	GACTTCTAGAAACAAAAACAAAATGGGCAGCAGCA	CAGTGGTACCACGAGTCGACATCCCCTTAGACCGA
	PmTFL1	AGTTTCTTTCTCACTCCTCATTCTC	GGAGGATGATGAAACACACTTGA
Rosa hybrida	RoFT	CCGGAATTCGCTACTAGCTGAGCAATAT	GTCGGTACCGAGAAAGACCCACAACT
	RoTFL1	CCGGAATTCCTCAAGGAAAACAGAAAAGGAT	GTCGGTACCAGCATCACTTGGTTGTTTATCTT
Fragaria ananassa	FaFT	GACTTCTAGACTCGGTGGCTTGTGTTTTCAATTTA	CAGTGGTACCACAACTCACAACTTACAAGCATTAT
	FaTFL1	CGGAATTCCTAATCCTTTCCCACAGTTTATCCA	GTCGGTACCTGCCTCCCTGCAAGGTGCCTAG
Photinia serrulata	PsFT	GCTCTAGACAAATAGCAATACAGCATGCAACGC	GCGGTACCTTATCTTCTCCTCCCTCCAGAGCCG
	PsTFL1	ACTCTCTTCTCTTCAGATGGCAA	TTTAGGTTATCGGGAAACAGGA
Pyracantha fortuneana	PfFT	GTTCCTTGGATACTTTGAGTTTTAGC	GCGGACTACATATATCTCTTTATCGTT
	PfTFL1	AAGAAGTGCTATTAGTTCCTCCTGA	TGGATGGAGGAGTTCTGGGTA
Spiraea cantoniensis	ScFT	ATGCCTAGGGATAGGGACCCTCTT	TTATCTTCTCCTCCCTCCAGAGCC
	ScTFL1	CCACACAAGGATACCAACATCTAAC	GATTTGGCTGTCCTAGCGTCTCC
Prunus yedoensis	PyTFL1	TCTGGAAACGATACCACTAAACTC	GCACGTTGCTCTCTCTTGATTGGAA
Prunus persica	PpFT	ATGCCTAGGGATAGGGACCCTCTT	CTTAATTATCTTCTCCTCCCTCCA

Table S4.
Gene specific primers used to construct expression plasmid.

Name	*Forward primer (5'-3')*	*Reverse primer(5'-3')*	*Enzyme restriction site*
RoFT	CCGGAATTCGCTACTAGCTGAGCAATAT	GTCGGTACCGAGAAAGACCCACAACT	EcoR1/Kpn1
RoFTmu1	GCTAGAGATCAGGAGCCTCTTGTTGTCGG	CCGACAACAAGAGGCTCCTGATCTCTAGC	
RoFTmu2	AGGGGGAGATGATCTTAGGATTTTCTACAC	GTGTAGAAAATCCTAAGATCATCTCCCCCT	
RoFTmu3	GAAACTCCACGGCCATCTATGGGGATCCATC	GATGGATCCCCATAGATGGCCGTGGAGTTTC	
RoFTmu4	TTTGCTGAGAACTGTAATCTTGGATCACCG	CGGTGATCCAAGATTACAGTTCTCAGCAAA	
RoFTmu5	TTTGCTGAGAACTATGATCTTGGATCACCG	CGGTGATCCAAGATCATAGTTCTCAGCAAA	
RoTFL1	CCGGAATTCCTCAAGGAAAACAGAAAAGGAT	GTCGGTACCAGCATCACTTGGTTGTTTATCTT	EcoR1/Kpn1
RoTFL1mu1	CCATATCTGAAGGAGTACTTGCACTGGATAG	CTATCCAGTGCAAGTACTCCTTCAGATATGG	
RoTFL1mu2	CCTCCTCCTTCCAAGCAGCACTTCGACAGTCG	CGACTGTCGAAGTGCTGCTTGGAAGGAGGAGG	
FaTFL1	CGGAATTCCTAATCCTTTCCCACAGTTTATCCA	GTCGGTACCTGCCTCCCTGCAAGGTGCCTAG	EcoR1/Kpn1
FaTFL1mu1	CCTTATTTGAAAGAGTACCTGCACTGGATTGT	ACAATCCAGTGCAGGTACTCTTTCAAATAAGG	
PhFT	CCGGAATTCGATAGTTCTTTATTGAGGTAGC	GTCGGTACCTACTAAGTTACATGATACGACGAC	EcoR1/Kpn1
PhFTmu1	GCTCCAGAAAATCGCCAGAATTTCAACAC	GTGTTGAAATTCTGGCGATTTTCTGGAGC	
AtFD	GCTCTAGAATGTTGTCATCAGCTAAGCATC	GGGGTACCCATGAGACAATCTCAATCCCCA	Xba1/Kpn1

Table S5.
Gene specific primers used for RT-PCR analysis.

Name	*Forward primer (5’-3’)*	*Reverse primer(5’-3’)*
NtEF1α	GAGGCACTTCCTGGTGACAAT	GGGCTCCTTCTCAATCTCCTTAC
RoFT	GGACTTTCTACACTCTGGTCTTGGT	CTTTGGCAGTTATAATAGACAGCGG
PmFT	TCACTTACGGTTTGAAGGAGGT	CATACACTGTTTGCCTACCCA
FaFT	TCAGGAGCCTCTTGTCGTCG	TAGCTCAAAATCTCTTGGCCGAAG
FaTFL1	CGCCAAACCTAGAGTTGAGATTC	CACCAAGGTCGTTTTCGGCT
RoTFL1	CTATTTCCTTCCTCAGTAACCACGA	GCTCTGCTCATGTTGAATCTGC
PhFT	CAAGGGTTGAGGTTGGAGGAGA	CCACCAGTACCATTTTCCCTTTG
AtFD	CCTCATCATCATCTCCTTTACC	AGTGACCGTGGTGGAATCG
AtEF1α	CAAGATGGATGCCACTACCC	AGTGGGAGACGAAGGGGCT

Table S6.
Gene specific primers used for qRT-PCR analysis.

Name	*Forward primer (5’-3’)*	*Reverse primer(5’-3’)*	*Accession No.*
NtEF1α	TGGTGGTGACTTTTGGTCCCA	ACAAACCCACGCTTGAGATCC	D63396
NtSOC1	CTGTCAGTACCATCCGAGCACGA	TCCACTTGAGCCTTGTCTCTGTTGA	CAA53782
NtAP1	AAGGATCATCGCGGCAAC	GTATTGTTCGCTTCACGCTTAT	AF009126
NtNFL	AATGCCCCACTAAGGTAACAAATC	GTCAAGGCAATGAAGTGCGTAG	U16174
AtEF1α	GATTGACAGGCGTTCTGGTAAG	CAGTCTGCCTCATGTCCCTCAC	AT1G07920
AtAP1	AAATCCAGCATCCTTACATGCTCTC	CAGTTCGAGATCATTCCTCCTCATT	AT1G69120

Table S7.

Gene specific primers used to construct yeast two-hybrid and BiFC vectors.

Name	*Forward primer (5'-3')*	*Reverse primer(5'-3')*	*Enzyme restriction site*
AtFD+PGADT7	ACCTCATATGATGTTGTCATCAGCTAAGCATCAGA	CGGGATCCCATGAGACAATCTCAATCCCCA	Nde1/BamH1
AtFT+PGBKT7	ACCTCATATGATGTCTATAAATATAAGAGACCC	GCGTCGACATAAAGGAAGAAGCCATCTAAAGTC	Nde1/Sal1
AtTFL1+PGBKT7	CGGAATTCATGGAGAATATGGGAACTAGAGT	GCGTCGACGCGTTTGCGTGCAGCGGT	EcoR1/Sal1
RoFT/mu1/mu2/mu3/mu4/mu5+PGBKT7	CGGAATTCATGCCTAGGGCTAGAGATCG	GCGTCGACGACAACGCTATACTCTCCTTCCA	EcoR1/Sal1
FaTFL1+PGBKT7	CGGAATTCATGGCAAGAATGTCGGAACCTC	GCGTCGACCCTCCCTGCAAGGTGCCTAGC	EcoR1/Sal1
RoTFL1+PGBKT7	CGGAATTCATGTCAGATCCTCTTGTTGTTGG	GCGTCGACTAATTAGCGTCTTCTTGCAGCAG	EcoR1/Sal1
PhFT+PGBKT7	CGGAATTCATGGAAAGAGGAAGAGATA	GCGTCGACTACTAAGTTACATGATACGACGA	EcoR1/Sal1
AtFD+YN173	TCTCTCTCGAGCTTTCGCGAGCTCATGTTGTCATCAGCTAAGCA	CATGGTGGCGATGGATCTTCTAGAAAATGGAGCTGTGGAAGACC	
AtFT+YC155	TCTCTCTCGAGCTTTCGCGAGCTCATGTCTATAAATATAAGAGACCC	GGTACCGGATCCCTCGAGTCTAGAAAGTCTTCTTCCTCCGCAGC	
AtTFL1+YC155	TCTCTCTCGAGCTTTCGCGAGCTCATGGAGAATATGGGAACTAGAGT	GGTACCGGATCCCTCGAGTCTAGAGCGTTTGCGTGCAGCGGT	
RoFT/mu1/mu2/mu3/mu4/mu5+ YC155	TCTCTCTCGAGCTTTCGCGAGCTCATGCCTAGGGCTAGAGATCG	GGTACCGGATCCCTCGAGTCTAGATACTCTCCTTCCACCAGAGC	
FaTFL1+ YC155	TCTCTCTCGAGCTTTCGCGAGCTCATGGCAAGAATGTCGGAACC	GGTACCGGATCCCTCGAGTCTAGAGCGTCTTCTTGCTGCCGTT	
RoTFL1+YC155	TCTCTCTCGAGCTTTCGCGAGCTCATGTCAGATCCTCTTGTTGTTGG	GGTACCGGATCCCTCGAGTCTAGATAATTAGCGTCTTCTTGCAGCAG	
PhFT+ YC155	TCTCTCTCGAGCTTTCGCGAGCTCATGGAAAGAGGAAGAGATACT	GGTACCGGATCCCTCGAGTCTAGACATGATACGACGACCACCA	
AtFT+PET32a	GCGAGCTCATGTCTATAAATATAAGAGACCC	GCGTCGACATAAAGGAAGAAGCCATCTAAAGTC	Sac1/Sal1

Table S8.

Flowering phenotypes of regenerated T_0 transgenic tobacco lines harboring *AtFD*.

genotype	n	no. leaves on main stem at flowering	plant height at first flower bud (cm)	time between transformed plantlet regeneration and first flower bud (days)
Wt	5	30.2±1.3	134.4±11.1	170.6±4.4
35S::AtFD	20	25.4±2.5	135.3±9.6	115.8±11.9

Notes: n = number of independent plants analyzed. Other codes are the same as given in Table 1.
