# Supplementary material for: The Divergence of Flowering Time Modulated by FT/TFL1 Is Independent to Their Interaction and Binding Activities
Source: Front Plant Sci. 2017 May 8;8:697. doi: 10.3389/fpls.2017.00697 (PMC5421193; doi:10.3389/fpls.2017.00697)
Supplement: Supplementary file 2 [file Image_1.PDF]

Identity=92.09%

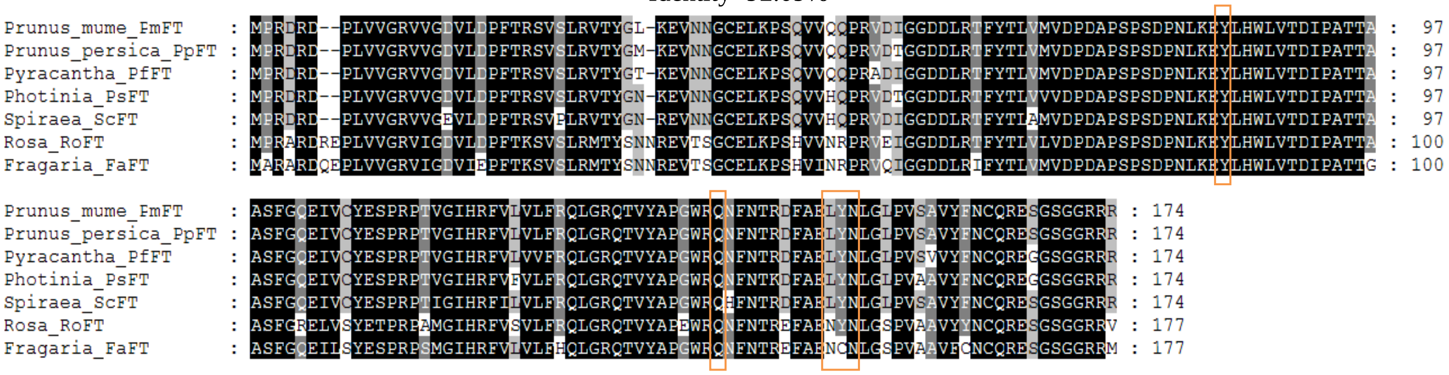

Identity=90.59%

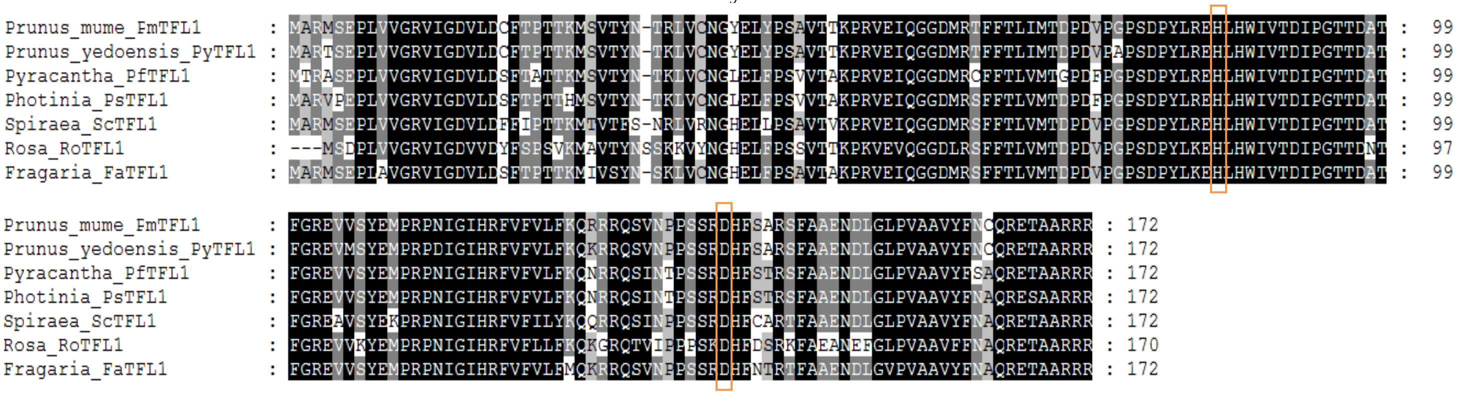

Supplementary Figure S1

Alignment of amino acid sequences of FT/TFL1 homolog of seven Rosaceae species including *Prunus mume* PmFT (CBY25181), *Rosa* RoFT (CBY25182), *Fragaria* FaFT (CBY25183), *Photinia* PsFT (AEO72028), *Pyracantha* PfFT (AEO72029), *Prunus persica* PpFT (AEO72030), *Spiraea* ScFT (AEO72031); *Prunus mume* PmTFL1 (AEO72021), *Rosa* RoTFL1 (AEO72022), *Prunus yedoensis* PyTFL1 (AEO72023), *Photinia* PsTFL1 (AEO72024), *Spiraea* ScTFL1 (AEO72025), *Pyracantha* PfTFL1 (AEO72026), *Fragaria* FaTFL1 (AEO72027). The Tyr85/His88 and Gln140/Asp144 residues which are likely the most critical residues for distinguishing FT and TFL1 activity are boxed. The analysis was performed using CLUSTALW MULTIPLE ALIGNMENT.
