# Supplementary material for: The Divergence of Flowering Time Modulated by FT/TFL1 Is Independent to Their Interaction and Binding Activities
Source: Front Plant Sci. 2017 May 8;8:697. doi: 10.3389/fpls.2017.00697 (PMC5421193; doi:10.3389/fpls.2017.00697)
Supplement: Supplementary file 3 [file Image_2.PDF]

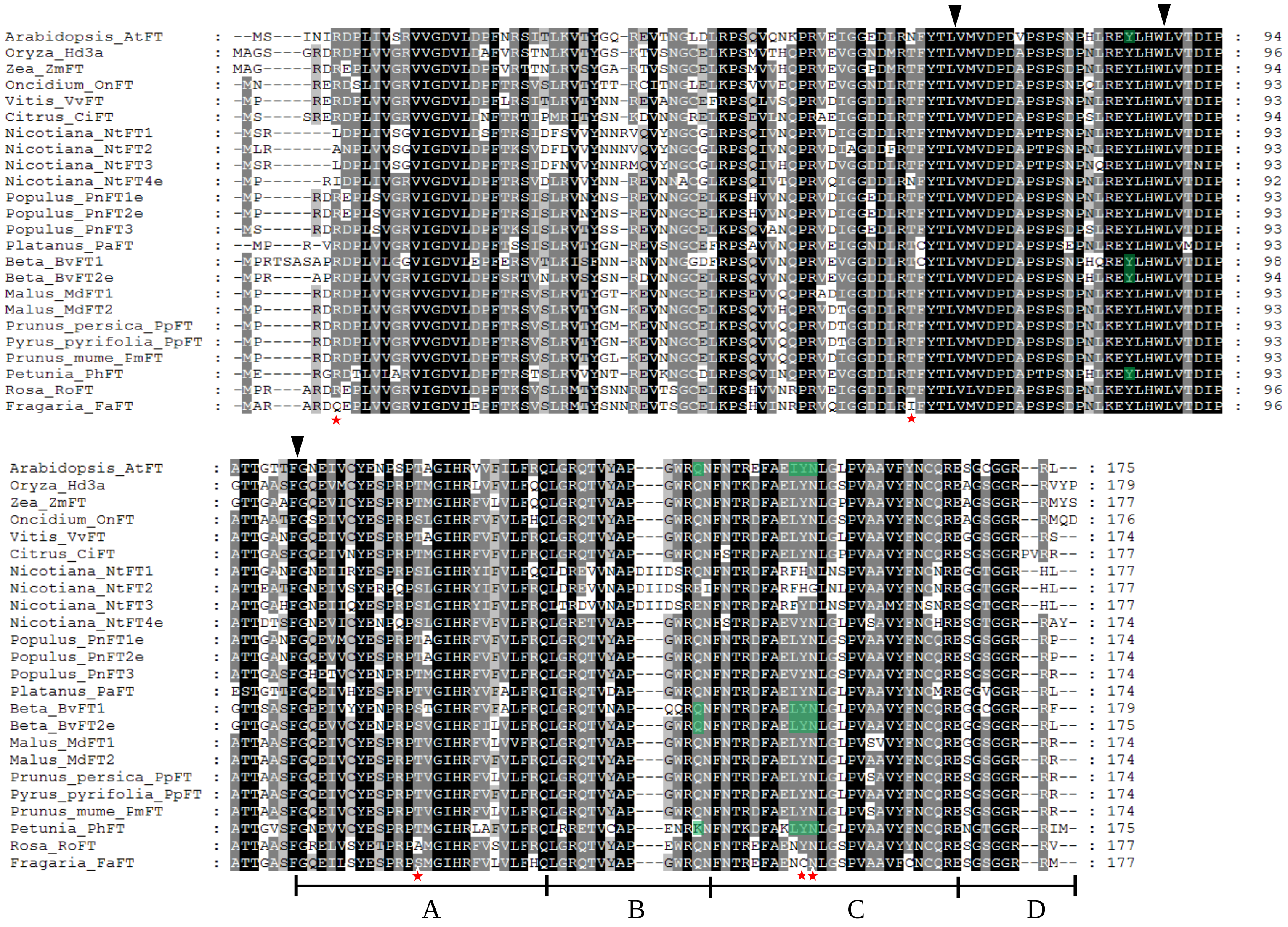

Arabidopsis_AtFT
Oryza_Hd3a
Zea_ZmFT
Oncidium_OnFT
Vitis_VvFT
Citrus_CiFT
Nicotiana_NtFT1
Nicotiana_NtFT2
Nicotiana_NtFT3
Nicotiana_NtFT4e
Populus_PnFT1e
Populus_PnFT2e
Populus_PnFT3
Platanus_PaFT
Beta_BvFT1
Beta_BvFT2e
Malus_MdFT1
Malus_MdFT2
Prunus_persica_PpFT
Pyrus_pyrifolia_PpFT
Prunus_mume_FmFT
Petunia_PhFT
Rosa_RoFT
Fragaria_FaFT
A
B
C
D

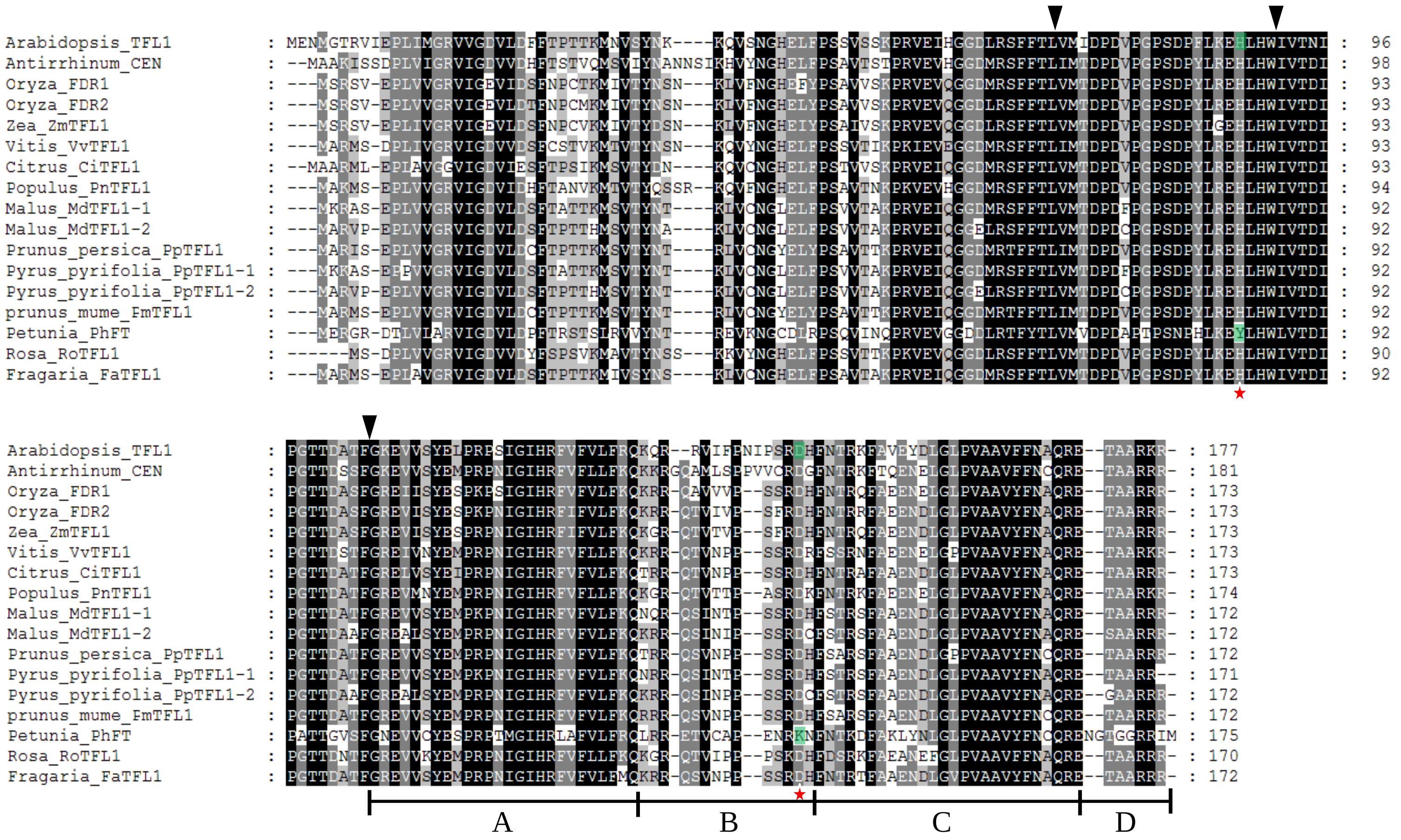

Supplementary Figure S2

The upper is the alignment of amino acid sequences of FT homologs including *Arabidopsis* AtFT (AF152096); *Beta* BvFT1 (HM448910); *Beta* BvFT2 (HM448912); *Citrus* CiFT (AB027456); *Fragaria* FaFT (CBY25183); *Malus* MdFT*1* (BAD08340); *Malus* MdFT2 (ADP69290); *Nicotiana* NtFT1 (JX679067); *Nicotiana* NtFT2 (JX679068); *Nicotiana* NtFT3 (JX679069); *Nicotiana* NtFT4 (JX679070); *Oncidium* OnFT (ACC59806); *Oryza* Hd3a (AB052944); *Petunia* PhFT (ADF42571); *Photinia* PsFT (AEO72028); *Platanus* PaFT (ACX34055); *Populus* PnFT1 (AB106111); *Populus* PnFT2 (AB109804); *Populus* PnFT3 (AB110612); *Prunus mume* PmFT (CBY25181); *Prunus persica* PpFT (AEO72030); *Pyracantha* PfFT (AEO72029); *Pyrus pyrifolia* PpFT (KF240775); *Rosa* RoFT (CBY25182); *Spiraea* ScFT (AEO72031); *Vitis* VvFT (ABF56526); *Zea* ZmFT (ABW96237);

The below is the alignment of amino acid sequences of TFL1 homologs including *Arabidopsis* TFL1 (U77674); *Antirrhinum* CEN (CAC21564); *Citrus* CiTFL1 (AY344245); *Fragaria* FaTFL1 (AEO72027); *Malus* MdTFL1-1 (AB162040); *Malus* MdTFL1-2 (AB366643); *Oryza* FDR1 (AF159883); *Oryza* FDR2 (AF159882); *Photinia* PsTFL1 (AEO72024); *Populus* PnTFL1 (AB181183); *Prunus mume* PmTFL1 (AEO72021); *Prunus persica* PpTFL1 (ADL62867); *Prunus yedoensis*

PyTFL1 (AEO72023); *Pyracantha* PfTFL1 (AEO72026); *Pyrus pyrifolia* PpTFL1-1 (BAD10962); *Pyrus pyrifolia* PpTFL1-2 (BAK74839); *Rosa* RoTFL1 (AEO72022); *Spiraea* ScTFL1 (AEO72025); *Vitis* VvTFL1 (AF378127); *Zea* ZmTFL1 (ABI98712).The black and gray areas indicate identical and similar amino acid, respectively. Triangles represent the exon boundaries of FT and TFL1 of Arabidopsis. The four segments of the exon 4 are shown as A, B, C and D (Ahn et al. 2006). Asterisks represent residues mutated in this study. Different residues between PhFT and Arabidopsis FT/TFL1 are indicated by green highlighting.
