# Supplementary material for: The Divergence of Flowering Time Modulated by FT/TFL1 Is Independent to Their Interaction and Binding Activities
Source: Front Plant Sci. 2017 May 8;8:697. doi: 10.3389/fpls.2017.00697 (PMC5421193; doi:10.3389/fpls.2017.00697)
Supplement: Supplementary file 4 [file Image_3.PDF]

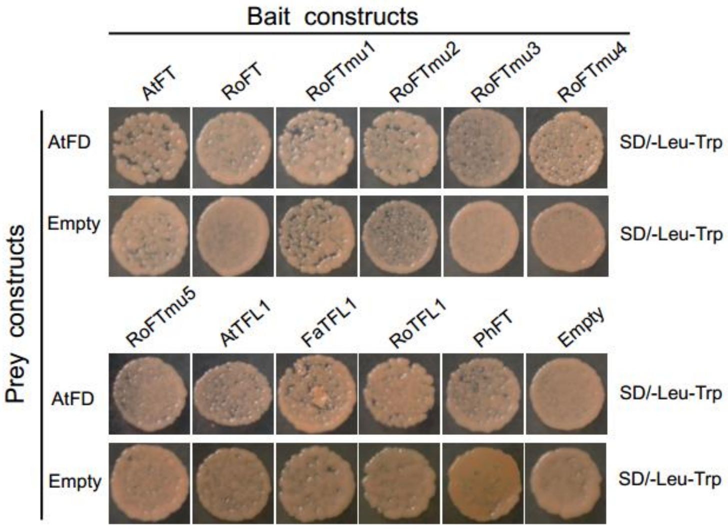


**Supplementary Figure S3**

Transformed yeast cells were grown on SD/-Leu-Trp selection medium in Yeast two-hybrid analysis.
