# Supplementary material for: The Divergence of Flowering Time Modulated by FT/TFL1 Is Independent to Their Interaction and Binding Activities
Source: Front Plant Sci. 2017 May 8;8:697. doi: 10.3389/fpls.2017.00697 (PMC5421193; doi:10.3389/fpls.2017.00697)
Supplement: Supplementary file 5 [file Image_4.pdf]

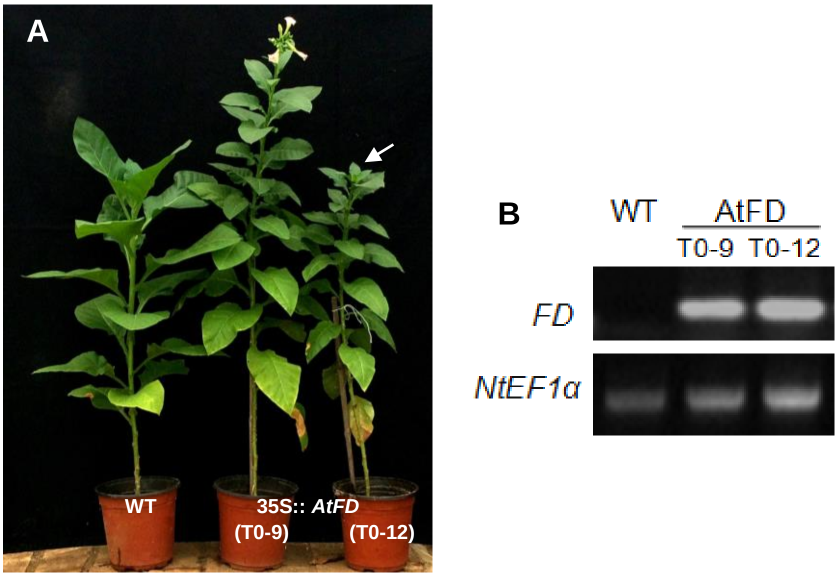

Supplementary Figure S4

Phenotype of transgenic tobacco plants harboring *Arabidopsis* FD (AtFD). (a) Transgenic tobacco lines containing *AtFD* after growth for 3 months in soil. (b) RT-PCR analysis to confirm the transgenic tobacco lines.
